# Continuous Biosensor Based on Particle Motion: How Does the Concentration Measurement Precision Depend on Time Scale?

**DOI:** 10.1021/acssensors.4c01586

**Published:** 2024-08-21

**Authors:** Rafiq M. Lubken, Yu-Ting Lin, Stijn R. R. Haenen, Max H. Bergkamp, Junhong Yan, Paul A. Nommensen, Menno W. J. Prins

**Affiliations:** † Helia Biomonitoring, Eindhoven 5612 AR, The Netherlands; ‡ Avebe Innovation Center, Groningen 9747 AA, The Netherlands; § Department of Biomedical Engineering, Eindhoven University of Technology, Eindhoven 5612 AZ, The Netherlands; ∥ Department of Applied Physics, Eindhoven University of Technology, Eindhoven 5612 AZ, The Netherlands; ⊥ Institute for Complex Molecular Systems (ICMS), Eindhoven University of Technology, Eindhoven 5612 AZ, The Netherlands

**Keywords:** continuous biosensing, continuous monitoring, affinity-based sensing, measurement precision, analytical performance

## Abstract

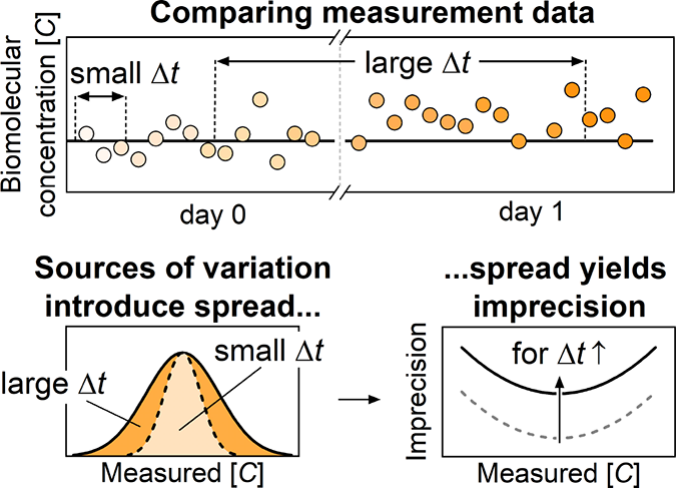

Continuous biosensors measure concentration–time profiles of biomolecular substances in order to allow for comparisons of measurement data over long periods of time. To make meaningful comparisons of time-dependent data, it is essential to understand how measurement imprecision depends on the time interval between two evaluation points, as the applicable imprecision determines the significance of measured concentration differences. Here, we define a set of measurement imprecisions that relate to different sources of variation and different time scales, ranging from minutes to weeks, and study these using statistical analyses of measurement data. The methodology is exemplified for Biosensing by Particle Motion (BPM), a continuous, affinity-based sensing technology with single-particle and single-molecule resolution. The studied BPM sensor measures specific small molecules (glycoalkaloids) in an industrial food matrix (potato fruit juice). Measurements were performed over several months at two different locations, on nearly 50 sensor cartridges with in total more than 1000 fluid injections. Statistical analyses of the measured signals and concentrations show that the relative residuals are normally distributed, allowing extraction and comparisons of the proposed imprecision parameters. The results indicate that sensor noise is the most important source of variation followed by sample pretreatment. Variations caused by fluidic transport, changes of the sensor during use (drift), and variations due to different sensor cartridges and cartridge replacements appear to be small. The imprecision due to sensor noise is recorded over few-minute time scales and is attributed to stochastic fluctuations of the single-molecule measurement principle, false-positive signals in the signal processing, and nonspecific interactions. The developed methodology elucidates both time-dependent and time-independent factors in the measurement imprecision, providing essential knowledge for interpreting concentration–time profiles as well as for further development of continuous biosensing technologies.

Continuous biosensors are being developed for monitoring applications in various fields, including fundamental biological research,^1−3^ healthcare,^4−11^ biotechnology,^12−17^ and environmental monitoring.^18−21^ A common aspect of continuous biosensor applications is the need to compare and interpret measurement data recorded at different time points, which requires insights into the time dependencies of the analytical performance. Traditional analytical parameters such as the coefficient of variation, the limit of detection, the limit of quantification,^22^ and absolute relative deviations,^23,24^ were not designed to reveal time-dependencies of continuous biosensors. Consequently, there is a need for analysis frameworks that can capture time-dependent sources of variation in a continuous biosensor.

In this work, we develop a framework to examine how the imprecision of a continuous biosensor depends on the time scale over which data points are compared. Experiments are designed and imprecision parameters are defined that span a wide range of time scales (minutes to weeks) and that encompass different sources of variation (both time-dependent and time-independent). The analysis framework is demonstrated using Biosensing by Particle Motion (BPM), a continuous, affinity-based sensing technology with single-particle and single-molecule resolution.^25−27^ This sensing technology is suitable for continuous monitoring since the molecular interactions are reversible, signals are recorded continuously, and the sensor uses the same biosensing materials (particles, antibodies, and conjugates) throughout its operation.

The BPM sensor studied in this paper has been developed to monitor glycoalkaloid (GA) molecules in a food matrix (potato fruit juice), relevant for real-time industrial process monitoring and control.^28^ We investigate how distributions observed in the measurement results are related to different sources of variation. Statistical analyses are applied to measurement data collected over approximately four months, during which the GA sensor system was used at two geographically distinct locations and operated by different persons. The data comprises measurements on nearly 50 cartridges, used on an equal number of days, with in total more than 1000 fluid injections and several measurements per fluid injection. The analyses provide insights into the contributions of various sources of variation to the measurement imprecision of the sensor system. This knowledge is essential for meaningful interpretation of observed concentration–time profiles and for guiding the further development of the continuous biosensing technology.

## Results and Discussion

### Measurement Imprecision and Sources of Variation across Different Time Scales

**Figure 1:**
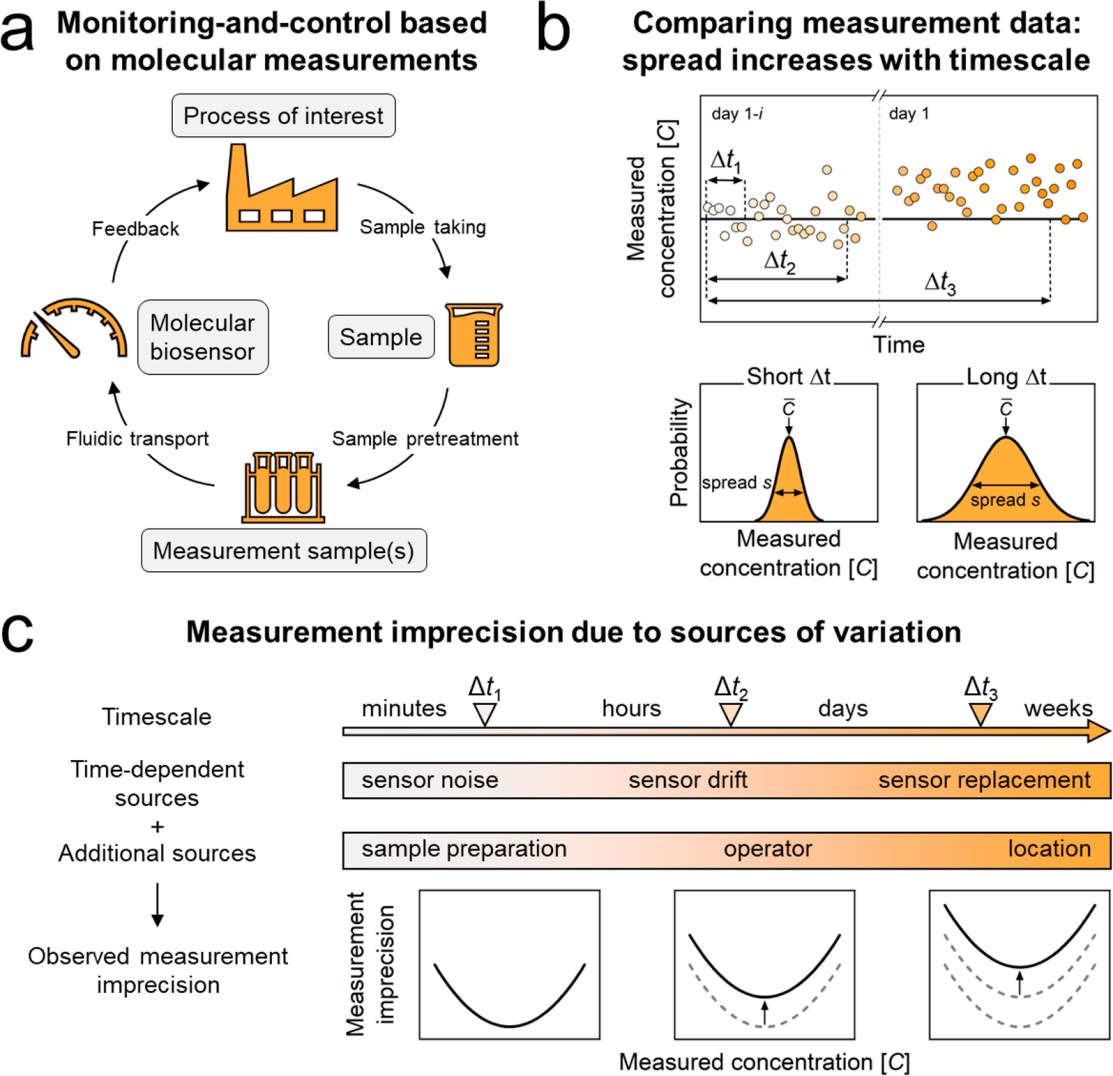
Comparison of data points collected over different time scales for monitoring and control. (a) Monitoring-and-control loop using molecular concentration data. Samples are taken from a process of interest and undergo sample pretreatment to produce measurement samples. The measurement samples are transported by a fluidic transport system into the molecular biosensor, where the molecular concentration is quantified and reported as feedback for optimizing the process. (b) Mock data of a true concentration–time profile in the process of interest (black line) and corresponding measurement data (dots with an white-to-orange time gradient). Variations in the measurement data over different time scales are visible, with periods *Δt*_1_ < *Δt*_2_ < *Δt*_3_. Long periods result in the inclusion of more sources of variation, resulting in a larger spread *s* in the measured concentration [*C*]. (c) Time-dependent and time-independent sources of variation both contribute to the observed measurement imprecision. For short periods, only a few sources of variation contribute as random errors, while for longer periods, many sources of variation contribute as random errors, leading to a higher measurement imprecision. The measurement imprecision is defined as the relative spread *s*/[*C̅*], where [*C̅*] is the mean of repeated concentration measurements.

The concept of measurement imprecision related to sources of variations across different time scales is illustrated in Figure 1. Figure 1a visualizes a continuous monitoring-and-control feedback loop for a process of interest, based on molecular concentration data measured using a molecular biosensing system. Samples are drawn at key positions in the process to provide concentration data that can be used to optimally control the process. The samples are pretreated, such as by filtration or dilution, resulting in measurement samples that are injected into the biosensor. From each sample drawn from the process, multiple measurement samples can be prepared, e.g., measurement samples at different dilutions. These measurement samples are injected into the biosensor, where the concentration of a specific molecular analyte is determined. The resulting concentration data are used to provide feedback, to control the process of interest, and to optimize its settings. This requires correct interpretations of the measurement data and therefore understanding of the origins of variations in the measurements.

When samples are drawn from a process at different time points, differences in the measurement results can on the one hand be caused by true analyte concentration differences and on the other hand by variations in the measurements themselves. This study quantifies variations in the measurements themselves, with the aim to understand the origins of spread in the data. Figure 1b sketches a time profile of the true concentration in a process of interest (black line) alongside measured concentration data points (dots with an white-to-orange time gradient) over two consecutive days. The recorded measurement data can be compared over different time scales, where Δ*t*_1_ < Δ*t*_2_ < Δ*t*_3_. When comparing measurement data recorded within a short period (Δ*t*_1_), the observed spread *s* in the measured concentration [*C*] is small. Comparing measurement data recorded over a long period (Δ*t*_3_), the observed spread in the measured concentration is larger, because more sources of variation affect the measurement data. In this paper, the concentration measurement imprecision is expressed as a relative spread *s*/[*C̅*], where [*C̅*] is the mean of the measured concentration data from repeated measurements. The concentration imprecision tends to increase when comparing measurement data recorded over prolonged periods, as more and more sources of variation play a role in the measurement.

Figure 1c illustrates how different sources of variation, both time-dependent and time-independent, influence the spread in the measured concentrations and thus the measurement imprecision. Over short periods (Δ*t*_1_), sensor noise predominantly contributes to the measurement imprecision, since on this time scale the sensor noise contributes as a random error. As the comparison period lengthens, more sources of variation contribute as random errors. For instance, when comparing measurement data over multiple hours (Δ*t*_2_), both minute-scale and hour-scale sensor variations show up as random errors. When comparing measurement data over multiple days (Δ*t*_3_), variations due to, for instance, sensor replacement may contribute as a random error as well, since multiple cartridges are used in this period. Besides time-dependent sources of variation, time-independent effects, such as variations due to different operators or due to different measurement locations, can play a role in the measurement results. Consequently, the total observed measurement imprecision is influenced by the period over which the measurement data points are collected, because more and more sources of variation, either time-dependent or time-independent, are included and result in an increasingly higher measurement imprecision.

### BPM Sensor for Glycoalkaloid Quantification

**Figure 2:**
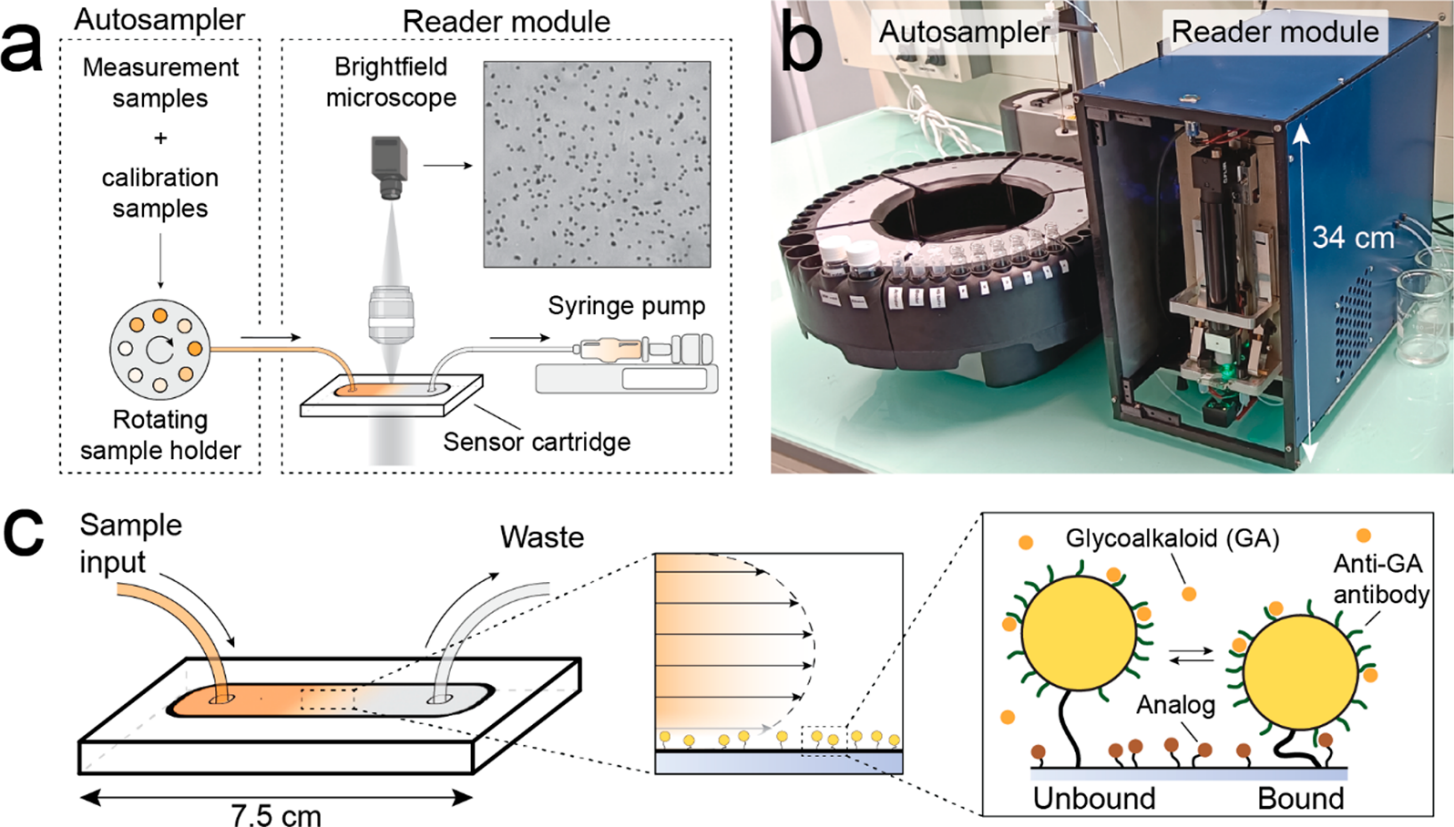
Continuous BPM sensor system for quantification of GA concentrations. (a) Schematic overview of the GA sensor components, i.e., an autosampler and a reader module. The autosampler module features a rotating sample holder containing calibration and measurement samples for automatic calibration and measurement cycles. The reader module includes a sensor cartridge, a small brightfield microscope, and a buret pump for fluid transport from the autosampler to the sensor cartridge. An integrated computer controls the autosampler, brightfield microscope, and buret pump, and performs image processing and sensor data analysis. (b) Photograph of the BPM sensor setup with the autosampler on the left and the reader module on the right. The dimensions of the reader module are 20 cm × 37 cm × 34 cm (w × l × h). (c) Sensor cartridge with an inlet for sample fluid and a waste outlet. Samples are supplied using advective transport (black arrows and black dashed parabola). The measurement principle of BPM for GA quantification^28^ utilizes a sensing surface to which micrometer-sized particles (yellow) are tethered with a double-stranded DNA molecule (black). The particles are functionalized with anti-GA antibodies (green) and the planar surface with GA-analog molecules (brown). The anti-GA antibodies on the particle reversibly bind to GA-analog molecules on the surface, causing the particles to switch between unbound and bound states. The switching behavior is continuously recorded by the brightfield microscope. When GA molecules are present in solution (orange), they can reversibly bind to the anti-GA antibodies, inhibiting the binding of the particles to the GA-analog molecules. Consequently, the motion behavior of the particles reflects the concentration of GA molecules in solution.

The statistical analyses to study sensor imprecision are exemplified for a continuous sensor based on BPM.^25−28^Figure 2 visualizes the sensor setup and the BPM sensor technology for measuring GA concentrations in potato fruit juice. GAs are a family of small-molecule compounds that occur naturally in potatoes. The molecules have a bitter taste and their concentration needs to be controlled in potato protein production processes.^29^Figure 2a schematically shows the components of the GA sensor. The GA sensor includes an autosampler and a reader module. The autosampler contains calibration samples and measurement samples, positioned on a rotating sample holder that enables automatic calibration of the sensor and automatic measurement of up to 36 measurement samples in a single run. The reader module contains a small brightfield microscope that records video images of particles inside a sensor cartridge. These particles scatter light strongly, allowing precise determination of their position and motion. The calibration samples and measurement samples in the autosampler are transported to the sensor cartridge by a buret pump and subsequently measured using the particle-based BPM sensing technology. A computer in the reader module performs the sensor data analysis and controls the autosampler, the brightfield microscope and the buret pump. A photograph of the GA sensor testing system is shown in Figure 2b.

Figure 2c sketches the sensor cartridge, where fluid (calibration sample or measurement sample) is injected, replacing the previous fluid via advective transport (the flow direction is sketched from left to right). GA molecules in the newly added fluid interact with the particles and with the biosensing surface in the cartridge. The cartridge is provided with particles that are coupled to the surface with a flexible double-stranded DNA tether. The sensing functionality of the particles originates from anti-glycoalkaloid (anti-GA) antibodies on the particles and glycoalkaloid-analog (GA-analog) molecules on the surface.^28^ In the absence of GA molecules in solution, the particles transiently bind to the surface due to antibody–analog interactions, causing the particles to switch between unbound and bound states. The frequency of switching is reduced when GA molecules are present in the solution, as these block the antibodies and therefore reduce the chance of particles binding to the biosensing surface. The sensor can operate continuously since (i) the molecular interactions are reversible due to the short-lived bonds between GA molecules and the anti-GA antibodies, (ii) the particle motion can be continuously recorded, and (iii) the functional materials in the BPM sensor (particles, anti-GA antibodies, and GA-analog) are not replaced and do not need to be replaced during sensor operation.

### Definition of Imprecision Parameters

Multiple sources of variation can contribute to the observed measurement imprecision. Here, we define a set of imprecision parameters, each encompassing one or more selected sources of variations, as shown in Table 1. The imprecision parameters (see top row with gray shading) relate to different sources of variation (see left column) over various time scales. The green shading indicates which sources of variation are included for each imprecision parameter, while no shading indicates exclusion. The time scale associated with an imprecision parameter represents the period over which the imprecision value can be used to compare data points; it is also the minimum period needed to experimentally obtain a quantitative value of the defined imprecision parameter.

**Table 1:**
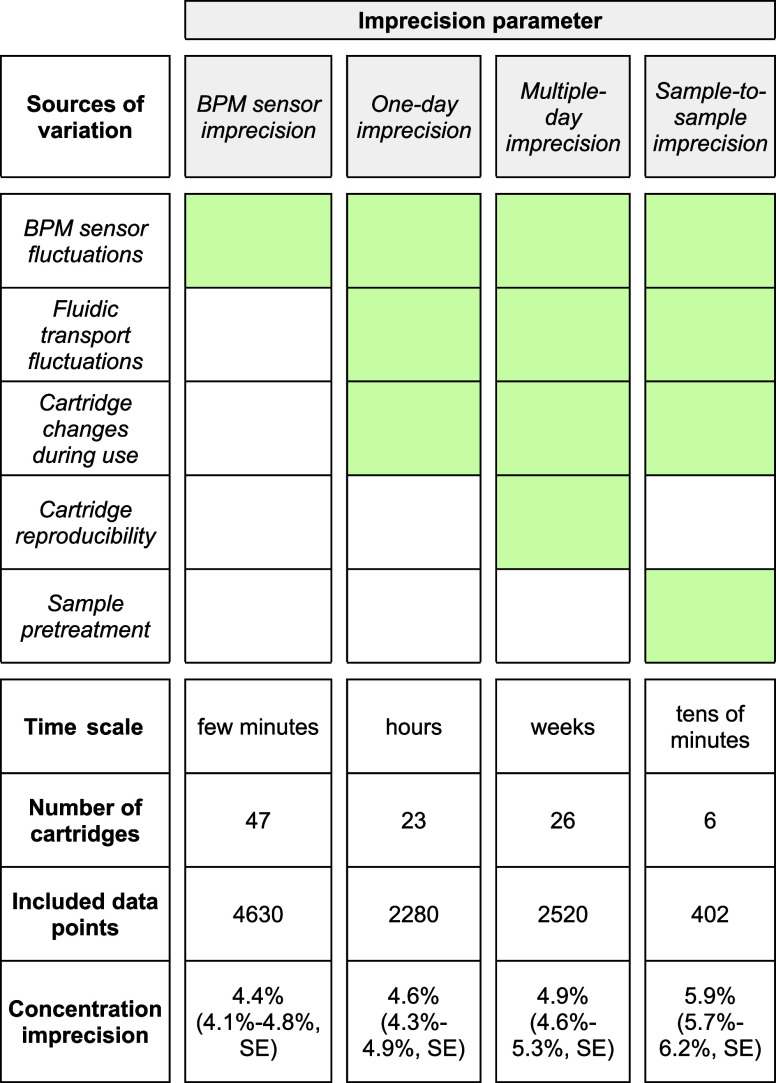
Definitions of Concentration Imprecision Parameters^a^

a The top row of the table, shaded in gray, lists the defined concentration imprecision parameters. The left column lists the sources of variation that are included in these parameters. Sources of variation included in the imprecision parameter are marked by green shading. The time scale of each imprecision parameter represents the period over which data points are compared. At the bottom of the Table, the rows provide details on the numbers of this study (the number of studied cartridges, the number of included data points) and the experimentally determined values for each concentration imprecision parameter. More details on the quantification of these concentration imprecision parameters are given in Figures 3 and 4.

In this study, the most fundamental imprecision is the BPM sensor imprecision, which includes variations caused by the BPM sensing principle itself, such as stochastic variations (e.g., binding/unbinding events, number of particles), sensor readout variations (e.g., mechanical/optical fluctuations), and variations in sensor data analysis (e.g., false positive/negative binding event detection).^26,30^ The one-day imprecision refers to the measurement imprecision when comparing data of the same sample measured over one day; measurement samples are measured multiple times on one sensor cartridge within a single day. This parameter includes variations due to fluidic transport and time-dependent changes in the sensor cartridge^31^ (see Supporting Information 1). The sample-to-sample imprecision refers to the measurement imprecision when comparing data measured on the same sample, but with separate sample pretreatment steps; measurement samples are measured on one sensor cartridge, including variations due to sample pretreatment steps (see also Figure 1a). Finally, the multiple-day imprecision refers to the measurement imprecision when comparing data from the same sample measured on multiple sensor cartridges over a period of days to weeks, which includes variations due to the use of different cartridges on different days. These defined imprecision parameters aid in understanding how time-dependent and time-independent sources of variation influence measurements performed by the continuous BPM sensor system.

### Quantification of the BPM Sensor Imprecision

Figure 3 illustrates the experimental process to determine the BPM sensor imprecision on a single sensor cartridge (see Figure 3a-c) and the resultant distribution of BPM sensor imprecisions for multiple cartridges (see Figure 3d). In the experiment of Figure 3a, eight consecutive calibration cycles were measured on a single cartridge. The top panel displays the sensor signal over time (gray dots), and the bottom panel shows the GA concentrations of the applied calibration samples (black line). The time sequence of samples, ranging in GA concentrations, is detailed in the inset of Figure 3b. On each sample, five measurements were performed. Measurements on calibration sample E serve to correct for signal drift over time (see Supporting Information 1). The 30 data points per calibration cycle (six calibration samples with five measurements per calibration sample) are used to fit a calibration curve. In Figure 3b, the mean (black dots, *N* = 5) and the corresponding sample standard deviation (black error bars) are depicted for calibration cycle 3 as an illustrative example. Since the BPM sensing principle is based on the occupancy of binder molecules by target molecules, the measured signal as a function of GA concentration should relate to the Langmuir isotherm. It was found that a Hill equation (black line) describes the measurement data well, using the sample standard deviations as weights in the fit. The Hill equation is expressed as follows:
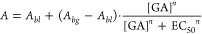
1where *A* is the time-corrected activity signal (i.e., data as presented in Figure 3a, cf. Supporting Information 1), *A*_*bl*_ the baseline signal which is the signal for [GA] → 0, *A*_*bg*_ the background signal which is the signal for [GA] → ∞, *n* the cooperativity factor or slope, and EC_50_ the halfway concentration at which the signal equals (*A*_*bg*_ + *A*_*bl*_)/2. The inset of Figure 3b shows the measured signal as a function of time, where the calibration sample sequence was E–A–B–C–D–E.

**Figure 3:**
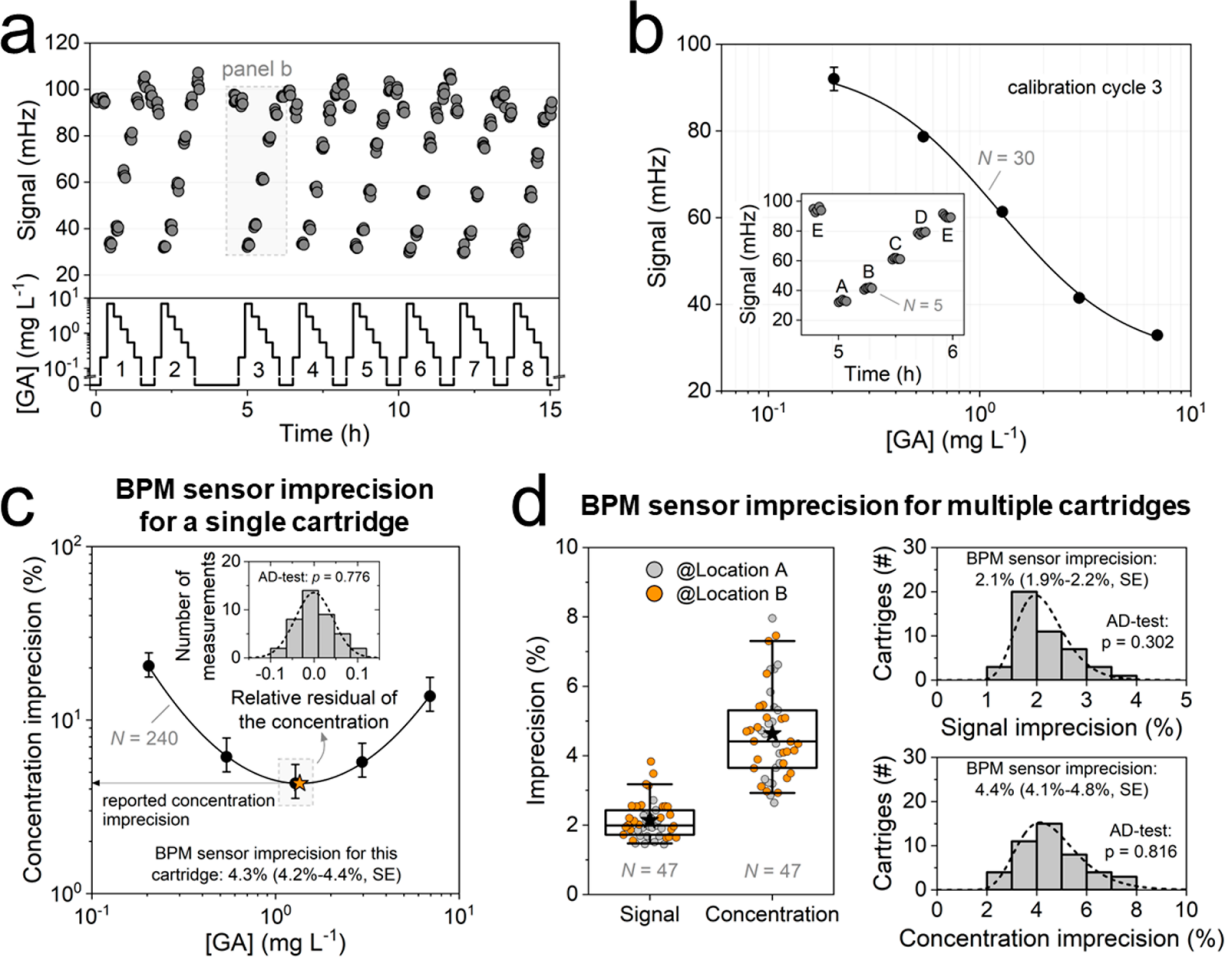
Quantification of BPM sensor imprecision for a single sensor cartridge and for multiple cartridges. (a) Switching activity signal of particles (mHz) as a function of time (hours, gray dots) for a GA concentration–time profile (black line) across eight sequential calibration cycles. Each calibration cycle includes the following calibration samples measured in sequence: 0, 0.20, 6.9, 3.0, 1.3, 0.54, 0.20, and 0 mg L^–1^. Each sample was measured five times consecutively. The blank samples (0 mg L^–1^) were used to monitor the baseline signal and were not used for the calibration curve. (b) Mean signal per calibration sample (mHz) as a function of GA concentration (mg L^–1^) (black dots, *N* = 5) for calibration cycle 3, with corresponding sample standard deviations (black error bars, mostly smaller than the symbol size). The GA concentrations of calibration samples are A = 6.9 mg L^–1^, B = 3.0 mg L^–1^, C = 1.3 mg L^–1^, D = 0.54 mg L^–1^, and E = 0.20 mg L^–1^ (see inset). Calibration sample E includes two measurement sets of five measurements each (see Supporting Information 1). The data were fitted using a Hill equation (see eq 1, black line), weighted by the sample standard deviations. For this calibration cycle, *A*_*bg*_ = 27 ± 4 mHz, *A*_*bl*_ = 96 ± 5 mHz, *n* = 1.5 ± 0.3, and *EC*_50_= 1.2 ± 0.1 mg L^–1^, with the errors representing the standard error of the fit. The inset shows the signal of all individual measurements over time for calibration cycle 3. (c) The mean concentration imprecision (%) as a function of GA concentration (mg L^–1^, black dots), with corresponding standard errors (black error bars) calculated using the data of all eight calibration cycles. The reported BPM sensor imprecision for this cartridge is 4.3% (4.2%–4.4%, SE for prediction), which is the minimum (orange star) of the second-order polynomial on a log–log scale (black line). This concentration imprecision curve is used as a reference curve in Figure 4a. The inset shows the histogram of the relative residuals of the concentrations measured on calibration sample C. The relative residuals follow a normal distribution (black dashed line) (*p* = 0.776, Anderson-Darling test). Data were collected at location A (see Materials and Methods). (d) BPM sensor imprecision across multiple cartridges: boxplot of the signal and concentration imprecision, with overlapping jitter plot representing individual cartridges measured at location A (gray) and at location B (orange), see Materials and Methods. The boxplot displays the mean (black star), 50th percentile (horizontal black line in box), 25th and 75th percentiles (top and bottom of box), and 5th and 95th percentiles (whiskers). Both signal and concentration BPM sensor imprecision follow a log-normal distribution (*p* = 0.302 and *p* = 0.816 respectively, Anderson-Darling test), with a signal imprecision at 2.1% (2.0%–2.1%, SE of the fit) and a concentration imprecision at 4.4% (4.3%–4.6%, SE of the fit), i.e., the median of the log-normal distribution. Data were measured at location A (21 cartridges) and location B (26 cartridges).

The measurement imprecision is quantified using the relative residuals of the measurement data, which can be either the relative residual of the measured signal or the relative residual of the measured concentration. The relative residual is the difference between the observed measurement result and the mean of a defined measurement set:
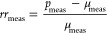
2where *rr*_meas_ is the observed relative residual of the signal or concentration within a defined measurement set according to Table 1, *p*_meas_ the measured signal or concentration, and μ_meas_ the mean of the measured signals or concentrations within the measurement set. It was shown that the histograms of the relative residuals of both signal and concentration are normally distributed (see Supporting Information 2). This means that measurement data can be taken together to increase the statistical power and obtain a more precise quantification of imprecision. Throughout this paper, the imprecision is defined as the sample standard deviation that results from fitting a relative residual histogram with a normal distribution.

The measurement sets for studying the relative residuals have to be chosen in agreement with the imprecision definitions in Table 1. For example, the BPM sensor imprecision refers to repeated measurements without fluid replacement, focusing on fluctuations occurring over a few minutes, while neglecting those occurring over longer time scales. A quantitative value of this imprecision can be determined by calculating the sample standard deviation of the relative residual distribution that results from eq 2. In the case of the BPM sensor imprecision, μ_meas_ represents the mean of the measured signals or concentrations of the five repeated measurements per calibration sample, see Figure 3a-b. This analysis assumes that the relative residuals of both signal and concentration are normally distributed and that the observed sample standard deviation does not depend on time or concentration (see Supporting Information 2).

Figure 3c illustrates the concentration imprecision of the BPM sensor as a function of GA concentration, determined from the eight calibration cycles shown in Figure 3a. Given the S-shaped calibration curve of the sensor (Figure 3b), the concentration imprecision as a function of GA concentration is expected to follow a second-order polynomial on a log–log scale for concentrations around the *EC*_50_, in case the signal error is independent of concentration and time (see Supporting Information 3). Indeed, the measurement data in Figure 3c show a concentration imprecision curve with a parabolic shape, with elevated imprecision values at both high and low analyte concentrations relative to the middle range. Near the *EC*_50_, where the calibration curve’s slope is steepest, the imprecision is lowest. The minimum imprecision (orange star) is determined using a second-order polynomial fit (black line), for which a total of 240 individual calibration measurements were used (i.e., eight calibration cycles, six calibration samples per cycle, five measurements per calibration sample). This minimum is referred to as the BPM sensor imprecision. For this example cartridge, the BPM sensor imprecision is 4.3% (4.2%–4.4%, SE for prediction). We verified that the BPM sensor imprecision quantified using calibration samples is comparable to a quantification using measurement samples (see Supporting Information 4). The inset shows the histogram of the relative residuals of all concentration measurements on calibration sample C. The assumption of normally distributed relative residuals of the concentration holds since the data is well described by a normal distribution (*p* = 0.776, Anderson-Darling test).

In Figure 3d, the BPM sensor imprecision is evaluated across multiple sensor cartridges. All measurement data recorded on a single cartridge are used to calculate the signal imprecision per cartridge. For the concentration imprecision, the minimum of the imprecision curve per cartridge (cf. Figure 3c) is reported. Between one and eight calibration cycles were measured per cartridge. Measurements were performed at location A (gray dots) and location B (orange dots), see Material and Methods. The data indicate that both the signal imprecision and the concentration imprecision across multiple cartridges follow a log-normal distribution (*p =* 0.302 and *p =* 0.816 for signal imprecision and concentration imprecision respectively, Anderson-Darling test). Using the fitted log-normal distribution, the signal imprecision is determined to be 2.1% (2.0%–2.1%, SE of the fit, assuming a log-normal distribution), and the concentration imprecision is 4.4% (4.3%–4.6%, SE of the fit, assuming a log-normal distribution), i.e., the median of the log-normal distribution. This suggests that the cartridge used as an example in Figure 3a-c is representative, as the BPM sensor concentration imprecision falls within the indicated confidence interval.

### Quantification of Other Concentration Imprecision Parameters

**Figure 4:**
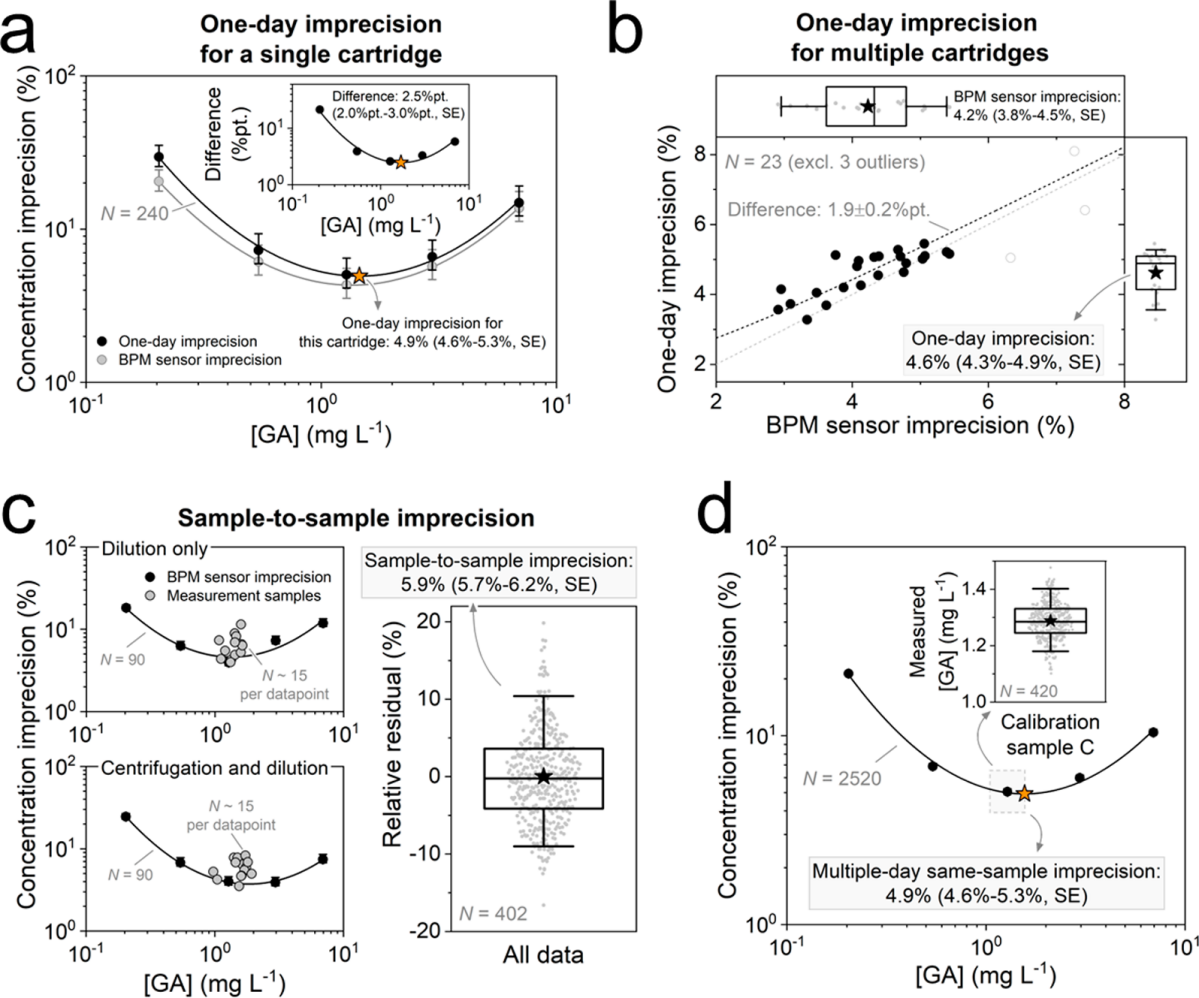
Quantification of one-day imprecision, sample-to-sample imprecision, and multiple-day imprecision, as defined in Table 1. (a) One-day imprecision for the cartridge of Figure 3a-c: the imprecision curve of the one-day imprecision (black dots) and BPM sensor imprecision (gray dots), with the corresponding standard error (black and gray error bars), as a function of GA concentration. The second-order polynomial fit (black line) of the one-day imprecision gives 4.9% (4.6%–5.3%, SE for prediction), which is the minimum of the fit (orange star). The inset shows the difference between the two imprecision definitions using eq 3. The fit (black line) of the absolute deviation gives 2.5% (2.0%–3.0%, SE for prediction), which is the minimum of the fitted curve (orange star). Data were measured at location A. (b) One-day imprecision for multiple cartridges (*N* = 23). The dependency between the observed BPM sensor imprecision and one-day imprecision per cartridge is fitted using eq 3, resulting in a difference of 1.9 ± 0.2%pt. (percent point, SE of the fit). Three outliers were omitted from the fit (gray open dots) due to signal drift in calibration measurements, leading to high BPM sensor imprecisions (data not shown here). A log-normal distribution fit of the BPM sensor imprecision for the selected cartridges results in 4.2% (3.8%–4.5%, SE of the fit), cf. Figure 3d. A log-normal distribution fit of the one-day imprecision for the selected cartridges results in 4.6% (4.3%–4.9%, SE of the fit). The boxplots indicate the mean (black star), 50th percentile (horizontal black line in box), 25th and 75th percentiles (top and bottom of box), and 5th and 95th percentiles (whiskers). Data were measured at location B. (c) Sample-to-sample variation: the concentration imprecision as a function of the mean measured concentration (gray dots), resulting from five individually prepared samples each for 15 measurement samples, considering the sample preparation step of dilution only (top) and centrifugation and dilution (bottom). The corresponding BPM sensor imprecision curves for the cartridges used for dilution only and for centrifugation and dilution (black dots), with the corresponding standard error (black error bars). Including all measured data (*N* = 402), the sample-to-sample imprecision was found to be 5.9% (5.7%–6.2%, SE of the fit), see inset. A correction was applied for the differences in BPM sensor imprecision per set of cartridges used for either centrifugation and dilution or for dilution only (see Supporting Information 5). The boxplot indicates the same features as described in panel b. Data were measured at location B. (d) Multiple-day imprecision including all selected cartridges in panel b (*N* = 26): imprecision curve of the multiple-day imprecision (black dots) with the corresponding standard error (black error bars, mostly smaller than the symbol size), as a function of GA concentration. The second-order polynomial fit (dashed black line) of the multiple-day imprecision gives 4.9% (4.6%–5.3%, SE for prediction), which is the minimum of the fitted curve (orange star). The inset shows a boxplot with all measured concentrations of calibration sample C (*N* = 420), measured on 26 cartridges during approximately two months. The boxplot indicates the same features as described in panels b and c. Data were measured at location B.

In Figure 4 the remaining concentration imprecision parameters, as detailed in Table 1, are quantified. We focus here on the concentration imprecision rather than the signal imprecision, because the concentration imprecision is most important for future applications. In Figure 4a, the one-day imprecision was quantified for a single cartridge (the same cartridge as in Figure 3a-c). The relative residuals of the concentration measurements were calculated per calibration sample, measured in all eight calibration cycles. For all GA concentrations, the one-day imprecision (black dots) is higher than the BPM sensor imprecision (gray circles), which is attributed to the additional sources of variation such as fluidic transport and cartridge changes during use (drift). A second-order polynomial fit (black line) yields a quantitative value of the one-day imprecision, which is 4.9% (4.6%–5.3%, SE for prediction), i.e., the minimum of the fit. The inset shows the difference between the one-day imprecision and the BPM sensor imprecision, where the difference was calculated using the following equation:
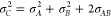
3where σ_*A*_ represents the BPM sensor concentration imprecision, σ_*B*_ the variation induced by the additional factors (e.g., fluidic transport and cartridge changes during use), σ_*C*_ the one-day imprecision, and σ_*AB*_ the covariance term. The inset in panel a assumes σ_*AB*_ = 0, i.e., no correlation between σ_*A*_ and σ_*B*_. The calculated σ_*B*_ is well-described by a second-order polynomial fit (black line), similar to the observed σ_*A*_ (Figure 3b) and σ_*C*_ (Figure 4a), suggesting that the signal variation underlying σ_*B*_ is independent of time and concentration, so σ_*AB*_ can be considered to be negligibly small (see Supporting Information 2). The second-order polynomial fit in the inset (black line) provides a quantitative value for the variation induced by the additional factors, which is 2.5%pt. (2.0%pt.−3.0%pt., SE for prediction), corresponding to the minimum of the fit (orange star).

Figure 4b illustrates the correlation between the one-day imprecision and the BPM sensor imprecision, as measured per cartridge. The two boxplots show the distributions of the BPM sensor imprecision and the one-day imprecision, both of which are log-normally distributed (*p =* 0.614 and *p =* 0.532 respectively, Anderson-Darling test). The BPM sensor imprecision is determined to be 4.2% (3.8%–4.5%, SE), and the one-day imprecision is 4.6% (4.3%–4.9%, SE) for the analyzed set of cartridges (*N* = 23). In the absence of differences between the two imprecision definitions, the data would align with the reference line *y* = *x* (gray dashed line). A deviation above the reference line suggests that additional sources of variation are included in the one-day imprecision. The measured cloud of points above the reference line confirms that the one-day imprecision includes more sources of variation than the BPM sensor imprecision, in agreement with the scheme in Table 1. Fitting the data with eq 3 (black dashed line), reveals an additional contribution of 1.9 ± 0.2%pt. (SE of the fit), which is consistent with the contribution found for the cartridge analyzed in Figure 4a. The data is well-described by eq 3 which indicates that there is a constant offset between the BPM sensor precision and the one-day imprecision. This offset across all observed BPM sensor imprecisions strongly suggests that the covariance term σ_*AB*_ is indeed negligibly small.

In Figure 4c the sample-to-sample imprecision is quantified, including variations due to dilution only (top) and due to both centrifugation and dilution (bottom). From each of the 14 potato fruit juice samples, five replicate measurement samples were prepared for each condition. Each replicate measurement sample was measured thrice, resulting in 15 concentration values per sample. The spread in the relative residuals and the mean concentration per sample are visualized in Figure 4c. For all samples, the observed concentration imprecision is comparable to, or larger than the BPM sensor imprecision of the cartridge used, due to additional sources of variation, such as sample pretreatment. No significant differences were observed between the concentration imprecision due to dilution and due to centrifugation and dilution (see Supporting Information 5). Consequently, all observed relative residuals are combined (see Figure 4c, right). Fitting a normal distribution to these relative residuals, the sample-to-sample variation was determined to be 5.9% (5.7%–6.2%, SE of the fit). Assuming fully independent sources of variation, the contribution of sample pretreatment can be determined using the imprecision of the one-day precision and the sample-to-sample imprecision using eq 3. The contribution of sample pretreatment variation was found to be approximately 3.6%pt.

In Figure 4d the multiple-day imprecision is quantified, using all calibration cycles measured on 26 cartridges at location B. The inset illustrates the measured GA concentration for calibration sample C as an example. A second-order polynomial fit (black line) provides a quantitative value for the multiple-day imprecision, determined to be 4.9% (4.6%–5.3%, SE for prediction), representing the minimum of the fit. Assuming fully independent sources of variation, the contribution of cartridge-to-cartridge variation can be determined using the one-day imprecision and the multiple-day precision using eq 3; the contribution of cartridge-to-cartridge variation was found to be approximately 1.7%pt.

## Conclusions

This paper focuses on the question how the concentration precision of a continuous biosensor depends on the time scale over which measurement data is compared. We defined a set of imprecision parameters encompassing different sources of variation across time scales ranging from minutes to weeks. The imprecision parameters and the analysis methodology were studied using a biosensor based on BPM, a continuous affinity-based sensing technology with single-molecule resolution that is suited for monitoring specific molecules, exemplified for glycoalkaloids in potato fruit juice. The BPM sensor is suited for continuous monitoring since the molecular interactions are reversible, the optical signal is recorded continuously, and the sensor uses the same biosensing materials throughout its operation.

Measurement data showed that the relative residuals of both the measured signal and the concentration are normally distributed. The primary sources of variations were identified to be the sensor noise (on a few-minute time scale) and the sample pretreatment (on tens of minutes time scale). Sensor noise is due to the stochastic nature of the sensor, signal processing, and nonspecific interactions, while variations from sample pretreatment are mainly due to a manual sample dilution step. Other sources of variation, such as fluidic transport processes, sensor changes during use (sensor drift), and reproducibility of the sensor cartridges (cartridge replacement), were found to be of minor importance.

Previous studies on the measurement precision of continuous biosensing technologies have mainly focused on single measurements,^23,24,32^ or on single concentration–time profiles,^33,34^ which do not reveal how precision depends on time. In this paper, we designed a conceptual analysis framework to quantify the concentration imprecision of continuous biosensors over a wide range of time scales, from minutes to weeks, including more and more sources of variation. This analysis provides insights into the contributions of various sources of variation to the measurement imprecision of the sensor system, essential for meaningful comparisons of concentration differences in concentration–time profiles.

Building on the findings presented in this paper, the next development steps will involve several aspects. First, the dominant sources of variability (sensor noise and manual sample dilution) can be addressed by increasing the number statistics and the specificity of switching events in the BPM biosensor, and by automating the sample handling. Second, it will be interesting to disentangle the underlying sources of variation of, for instance, stochastics, false-positive event detection, and nonspecific interactions,^26,30^ as these will inspire further work in sensor biochemistry (e.g., surface properties, coupling chemistries, antibody types, antibody stability, biomarkers, matrices) and in data acquisition and data analysis (e.g., optimizations of data acquisition times). Third, the presented methodology can be extended to study the time dependencies of more analytical performance parameters, e.g., measurement accuracy (measured concentrations versus a reference method), limit of detection, and limit of quantification. Assessing total concentration deviations, caused by both random and systematic variations, will provide deep insights into the functional properties of the sensor technology and its suitability for various applications. Lastly, the analysis framework introduced in this paper can be generalized and compared to simulations. Statistical simulation models, tested and iteratively improved using experimental data, will be able to clarify the limits of analytical performance that can be achieved with continuous biosensing technologies.

In summary, we expect that the developed methodology lays groundwork for future research aimed at elucidating time-dependent factors in the analytical performance of continuous biosensors and at guiding improvements that are necessary for applying continuous biosensing technologies in fields such as dynamic biological systems, biotechnological process control, and patient monitoring.

## Materials and Methods

### BPM Sensor Cartridges and Sample Preparations

The biomaterials, particles, and coupling methods used in GA sensor cartridges have been described by Vu et al.^28^ In this study custom-made injection molded cartridges from cyclic olefin copolymer were used. Fourteen potato fruit juice (PFJ) samples were collected from an industrial potato processing line and were stored at −20 °C. For preparing measurement samples, PFJ samples were thawed at room temperature and thereafter homogenized by vortexing. Thereafter, the samples were centrifuged at 6000 × *g* for 5 min using a tabletop spinner (Eppendorf MiniSpin) to spin down fibrous and aggregate materials, in order to prevent clogging of microfluidic tubing. Subsequently, the supernatant was aspirated and diluted in PBS with an additional 500 mM NaCl, in order to bring the GA concentrations in the measurement samples into a measurable range (50–200x dilution, see Supporting Information 6). The centrifuged and diluted samples were directly measured, or stored at −20 °C until further use. For calibration sample preparation, two PFJ samples (one with [GA] = 1416 ppm, and one with [GA] = 42 ppm, quantified by HPLC) were thawed, vortexed, and centrifuged as described above. Subsequently, the supernatant of both PFJ samples was aspirated and mixed in 5 different ratios (high-[GA]:low-[GA] → 100:0, 41:59, 16:84, 5:95, 0:100). Then the mixed samples were 200x diluted in PBS with an additional 500 mM NaCl. The centrifuged and diluted calibration samples were directly measured, or stored at −20 °C until further use.

### GA Sensor System and Measurements

The GA sensor system comprises an autosampler module (HTA) and a custom-built reader module. The reader module consists of a small optical microscope, multiway valves, a bubble detector, a buret pump (AMF) for fluidic transport, and a computer for controlling the optical system, fluidic transport, and data analysis. Prior to sample measurement, 100 μL of sample was transported through the cartridge at 100 μL/min in order to replace the fluid from the previous sample. Five sequential particle-motion measurements of 1 min each were performed per calibration sample. In experiments with measurement samples, three sequential particle-motion measurements of 1 min each were performed. Signal analysis and signal correction was performed as described in Supporting Information 1. Measurements were done at location A (Helia lab in Eindhoven) and at location B (Avebe QC lab in Gasselternijveen), see Supporting Information 7.

## Supporting Information Available

The following files are available free of charge The Supporting Information is available free of charge at https://pubs.acs.org/doi/10.1021/acssensors.4c01586.

Methods to correct the measured sensor signal, time and concentration dependencies of imprecision parameters, imprecision quantification, and comparison of sensor performance between two locations (PDF)
