# Supplementary material for: Continuous Biosensor Based on Particle Motion: How Does the Concentration Measurement Precision Depend on Time Scale?
Source: ACS Sens. 2024 Aug 21;9(9):4924–33. doi: 10.1021/acssensors.4c01586 (PMC11443519; doi:10.1021/acssensors.4c01586)
Supplement: Supplementary file 1 — se4c01586_si_001.pdf [file se4c01586_si_001.pdf]

Continuous Biosensor Based on Particle Motion: How Does the Concentration Measurement Precision Depend on Time Scale?

Rafiq M. Lubken[1], Yu-Ting Lin[1], Stijn R. R. Haenen[1], Max H. Bergkamp[1], Junhong Yan[1], Paul A. Nommensen[2], & Menno W. J. Prins[1,3,4,5*]

[1]Helia Biomonitoring, Eindhoven, 5612 AR, The Netherlands.

[2]Avebe Innovation Center, Groningen, 9747 AA, The Netherlands.

[3]Department of Biomedical Engineering, Eindhoven University of Technology, Eindhoven, 5612 AZ, The Netherlands.

[4]Department of Applied Physics, Eindhoven University of Technology, Eindhoven, 5612 AZ, The Netherlands.

[5]Institute for Complex Molecular Systems (ICMS), Eindhoven University of Technology, Eindhoven, 5612 AZ, The Netherlands.

*email: m.w.j.prins@tue.nl

Supporting Information

Table of Contents

1. Time-dependency and correction of the measured BPM sensor signal
2. Signal imprecision parameter and its dependencies
3. How the concentration imprecision depends on concentration
4. Quantifying BPM sensor imprecision using measurement samples
5. Sample-to-sample imprecision
6. Dilution series of PFJ samples
7. Comparison between measurements performed at locations A and B
8. References

1. Time-dependency and correction of the measured BPM sensor signal

Figure S1 shows the measured signal of a Biosensing by Particle Motion (BPM) sensor as a function of time. At equal glycoalkaloid (GA) concentrations, the signal gradually decreases over time. This time dependency is attributed to losses of biomolecular components in the sensor.[1] In this Section, we explain the signal correction method that is applied to correct for the observed time-dependency.

Figure S1 shows that the time-dependency of the signal can be described by a single-exponential decay function. To obtain a time-independent measurement signal, the rate at which the signal changes, *i.e.*, the decay rate k_{decay}, is quantified using the following equation:

$$A_{\mathrm{meas}} = A_{bg} + A_{\Delta} \cdot e^{-k_{\mathrm{decay}} t} \qquad \text{(S1)}$$

where A_{meas} is the measured signal, A_{bg} is the background signal that follows from the calibration curve (using uncorrected measurement data), A_{Δ} is the difference between the measured activity at $t = 0$ and at $t \to \infty$, k_{decay} the decay rate, and t the time at which the signal A_{meas} was measured. The measurement data of the 0.20 mg L^{-1} samples (gray dots with red outlines) were used to fit Equation S1 (dashed red line). The decay curve in Figure S1 shows that Equation S1 describes the time effect in the measurement data well. The fit parameter k_{decay} was used to apply a time correction to all data, using Equation S2:

$$A_{\mathrm{corr}} = A_{bg} + e^{k_{\mathrm{decay}} t} \cdot |A_{\mathrm{meas}} - A_{bg}| \qquad \text{(S2)}$$

where A_{corr} is the corrected signal. Since a quantitative value for the background signal is required in Equations S1-S2, first a calibration curve is fitted using the measured signal (*i.e.*, the signal that has not been corrected) from which the background signal is extracted (see Equation 1). Then k_{decay} is determined using Equation S1 and subsequently a time correction is applied using Equation S2. Then, a second calibration curve is fitted using the time-corrected signal; this second calibration curve is used to determine the concentration of subsequently measured samples. Throughout the paper, the time-corrected signal is referred to as the (measured) signal.

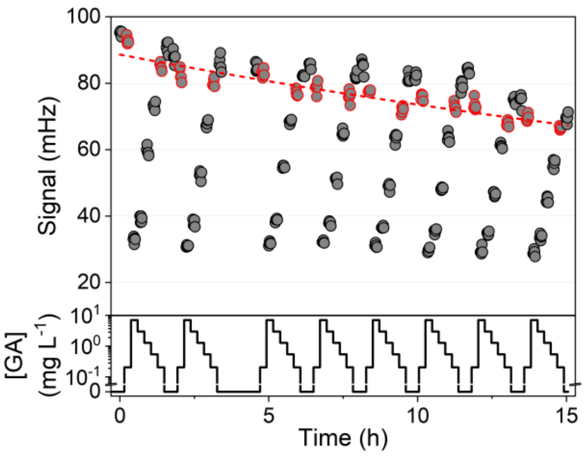

Figure S1. Time-dependency of the measured signal. Measured signal in mHz as a function of time in hours (gray dots) for a GA concentration-time profile (black line) of 8 sequential calibration cycles. In each calibration cycle, the following calibration samples were measured in the indicated order: 0, 0.20, 6.9, 3.0, 1.3, 0.54, 0.20, and 0 mg L^{-1}, where each calibration sample was measured five times. A single-exponential fit (red dashed line) through the measurement data of the calibration samples with 0.20 mg L^{-1} (red circles) was used to correct for the time dependence. For this example cartridge, it was found that A_{bg} = 23±3 mHz (from latest calibration curve fit), A_{Δ} = 65.0±0.6 mHz, and k_{decay} = (7.3±0.3) · 10^{-6} s^{-1}. The reported errors are the standard error of the fit. The time-corrected signal as a function of time for the same dataset is visualized in Figure 3a.

Since the calibration data of the lowest GA concentration (*i.e.*, 0.20 mg L^{-1}) was used for fitting Equation S1, the time correction mainly corrects for time effects in the baseline signal. Other parameters in Equation 1, such as the background signal, might change differently as a function of time. This is further investigated in Figures S2-S3.

Figure S2 shows plots revealing the time-dependencies of all (normalized) fit parameters in Equation 1 using uncorrected measurement data. It appears that there is a strong (linear) correlation between the baseline signal A_{bl} and time (r = -0.58, $p < 0.001$, Pearson correlation coefficient), while the correlations between the background signal A_{bg}, slope n, and EC_{50}, and time appear to be weak and not significant. So for correcting time effects, we conclude that a concentration should be used that relates to the baseline signal, *i.e.*, a low concentration. Moreover, A_{Δ} is large for low GA concentrations and therefore the decay rate could be determined with a smaller error of the fit.

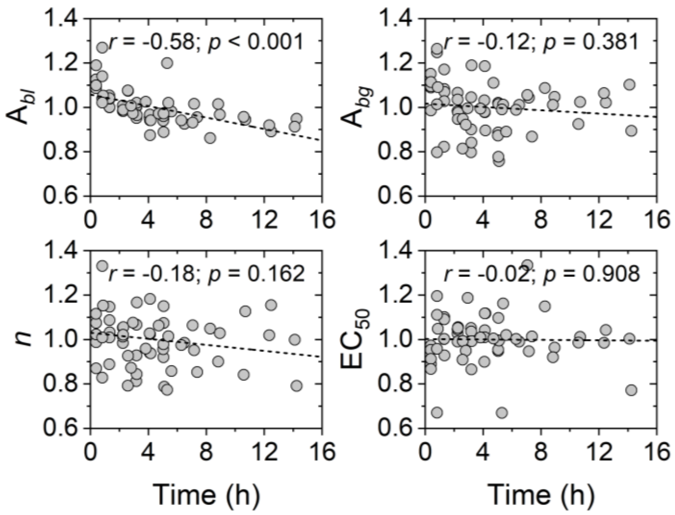

Figure S2. Time-dependencies of all fit parameters in Equation 1 using uncorrected measurement data. Correlation between normalized fit parameters baseline signal A_{bl}, background signal A_{bg}, slope n, and EC_{50}, and time, per calibration cycle (gray dots) measured on multiple cartridges using uncorrected measurement data. The found Pearson correlation coefficients r with corresponding p-values are given. To compare the fit parameters over time including multiple cartridges, the absolute values of the fit parameters are normalized per cartridge; there are between three and eight calibration cycles measured per cartridge. Data were measured on locations A and B.

In follow-up work, we will further study the influence of time corrections on time-dependencies of fit parameters, in order to optimize the correction method toward the smallest possible time effects.

2. Signal imprecision parameter and its dependencies

A measurement imprecision can be defined using different parameters, such as a distribution of a standard deviation or a distribution of a residual, expressed either as an absolute or as a relative parameter.

The use of a distribution of a standard deviation to express the imprecision has several disadvantages:

1. The standard deviation per measurement set is not normally distributed, because a standard deviation can only assume positive values. Alternatively, the distribution of a parameter with only positive values can be approximated by a log-normal distribution.

2. A standard deviation cannot be determined reliably from a small measurement set. The standard deviation of a small measurement set depends on the sample size and has a wide confidence interval for small sample sizes.

3. A standard deviation gives deviations when a drift is present in the signal. Combining measurement sets measured at different time points would result in an overestimated standard deviation, since the mean of the distribution drifts over time. For BPM, an underlying signal drift is expected, for instance, due to time-dependent losses of biochemical components and corresponding signal correction methods (see Supporting Information 1).

Preferably, the imprecision is quantified using a parameter that is normally distributed, that has narrow confidence intervals, and that is not sensitive to drift in the signal. Such properties will allow that large data sets can be taken into account in order to increase statistical power and obtain reliable quantifications of imprecision.

In this Section, both the standard deviation and the relative residual are investigated as parameters to quantify imprecision, in order to support the choice of this paper to quantify imprecision by the distribution of relative residuals.

(Relative) standard deviation of the signal

In Figure S3, using the measurement data of the example cartridge of Figure 3, the standard deviation (Figure S3a) and relative standard deviation (Figure S3b) calculated per calibration sample (each comprising five repeated measurements) are visualized as a function of calibration sample, using the measurement data of all calibration cycles. The (relative) standard deviations calculated from the first and second calibration sample E are not significantly different (data not shown here) and therefore these two measurement sets are combined into a single measurement set. The *p*-values given in Figure S3 result from a one-way ANOVA on the mean of the (relative) standard deviation per calibration sample per calibration cycle, assuming a log-normal distributed (relative) standard deviation.

The left panel of Figure S3a shows a boxplot of the standard deviation per calibration sample measurement. In this Figure, the mean (black star), the 25th and 75th percentiles (black box), 50% percentile (horizontal black line in box), 5th and 95th percentiles (whiskers), and calculated standard deviation per calibration sample measurement (gray dots) are visualized. Considering all calibration samples, the hypothesis that the means of the calculated standard deviation are the same is rejected ($p = 0.007$); this indicates that there is a concentration-dependency of the standard deviation of the signal. The right panel of Figure S3a gives the *p*-value from all possible combinations of comparing the calibration samples; only for comparing

calibration samples A and E, and B and E a significantly different mean was observed (p = 0.019 and p = 0.042, respectively). However, a clear concentration-dependency is visible, since the box is shifting towards higher standard deviations for a decreasing concentration.

The left panel of Figure S3b shows a boxplot of the relative standard deviation per calibration sample measurement. Considering all calibration samples, as well as considering calibration samples B-E, the hypothesis that the means of the standard deviations are the same is not rejected (p = 0.175 and p = 0.418, respectively). This indicates that there is no significant concentration-dependency of the relative standard deviation. The right panel of Figure S3b gives the p-value from all possible combinations of comparing the calibration samples. In contrast to the standard deviation, the relative standard deviation does not show any combinations with a significantly different mean, though visually a concentration-dependency can be observed. Here, the relative standard deviation decreases for a decreasing concentration. This is as expected since the relative contribution of nonspecific interactions compared to the relative contribution of specific interactions is larger at high concentrations.

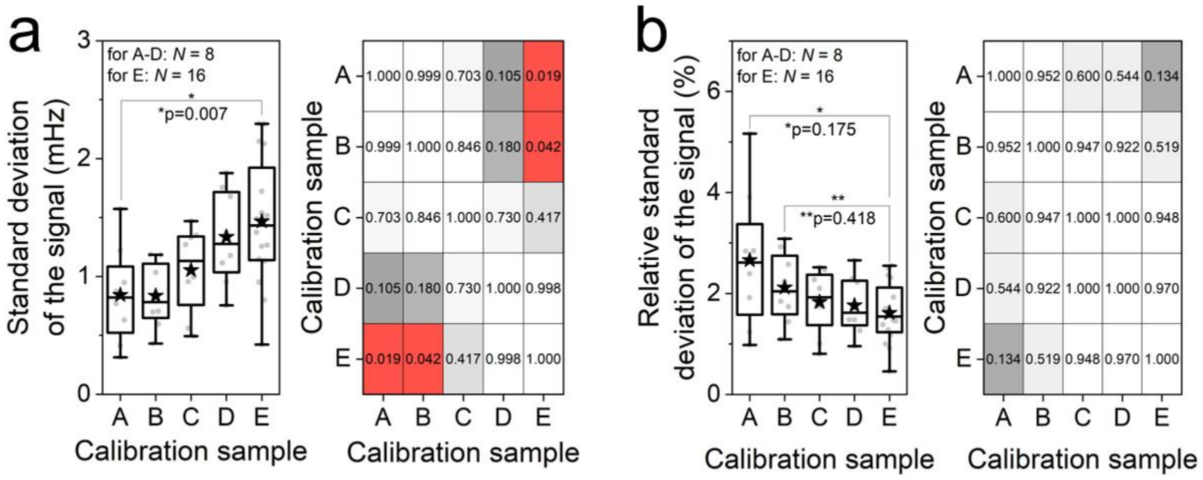

Figure S3. The (relative) standard deviation of the signal per calibration sample. (a) Boxplot of the calculated standard deviation of the measured signal in mHz per calibration sample. The boxplot indicates the mean (black star), 50th percentile (horizontal black line in box), 25th and 75th percentiles (top and bottom of box), and 5th and 95th percentiles (whiskers). The standard deviations per calibration sample per calibration cycle are visualized (gray dots), where N = 8 for calibration samples A-D, and N = 16 for calibration sample E. Considering all calibration samples, the hypothesis that the mean of the calculated standard deviation for each calibration sample comes from the same distribution can be rejected (p = 0.007). The matrix shows that there only exists a significant difference (red shading) between the means of calculated standard deviation between calibration samples A and E (p = 0.019), and B and E (p = 0.042). **(b)** Boxplot of the calculated relative standard deviation of the measured signal in mHz per calibration sample. The boxplot visualizes the same features as in panel a. Considering all calibration samples, the hypothesis that the mean of the calculated relative standard deviation for each calibration sample comes from the same distribution cannot be rejected (p = 0.175). Considering calibration samples A-D, the same conclusion can be drawn (p = 0.418). All reported p-values result from a one-way ANOVA, assuming a log-normal distributed (relative) standard deviation. An Anderson-Darling test indicates that a combined measurement set of all calibration samples can be assumed to be log-normal distributed (p = 0.421, data not shown here), rather than normally distributed (p = 0.064, data not shown here).

(Relative) residual of the signal

In Figure S4, using the measurement data of the example cartridge of Figure 3, the residual (Figure S4a) and relative residual (Figure S4b) calculated per calibration sample (each comprising five repeated measurements) are visualized as a function of calibration sample, using the measurement data of all calibration cycles. Again, the (relative) residuals calculated from the first and second calibration sample E are not significantly different (data not shown

here) and therefore these two measurement sets are combined into a single measurement set. The p-values given in Figure S4 result from a Bartlett's test on the variance of the (relative) residual per calibration sample per calibration cycle, assuming normally distributed (relative) residuals.

The left panel of Figure S4a shows a boxplot of the calculated residual per calibration sample measurement. In this Figure, the mean (black star), the 25th and 75th percentiles (black box), 50th percentile (horizontal black line in box), 5th and 95th percentiles (whiskers), and calculated residual per calibration sample measurement (gray dots) are visualized. Considering all calibration samples, the hypothesis that the variances of the calculated residual per calibration sample are the same is rejected (p < 0.001); this indicates that there is a concentration-dependency of the variance. The right panel of Figure S4a gives the p-value from all possible combinations of comparing the calibration samples. Similar trends are visible in Figure S3a: for increasing concentration, the variance of the residual (width of the residual distribution) becomes larger.

The left panel of Figure S4b shows a boxplot of the relative residual of all calibration samples. Considering all calibration samples, the hypothesis that the variances of the calculated residual per calibration sample are the same is rejected (p < 0.001). However, only considering calibration samples B-E yields a similar variance (p = 0.418); this indicates that there is no concentration-dependency of the relative residual. The right panel of Figure S4b gives the p-value from all possible combinations of comparing the calibration samples. It appears that calibration sample A has a significantly different variance compared to other calibration samples. Again, similar trends are visible in Figure S3b: for increasing concentration, the variance of the relative residual (width of the relative residual distribution) becomes smaller as the relative contribution of nonspecific interactions compared to the relative contribution of specific interactions is larger at high concentrations.

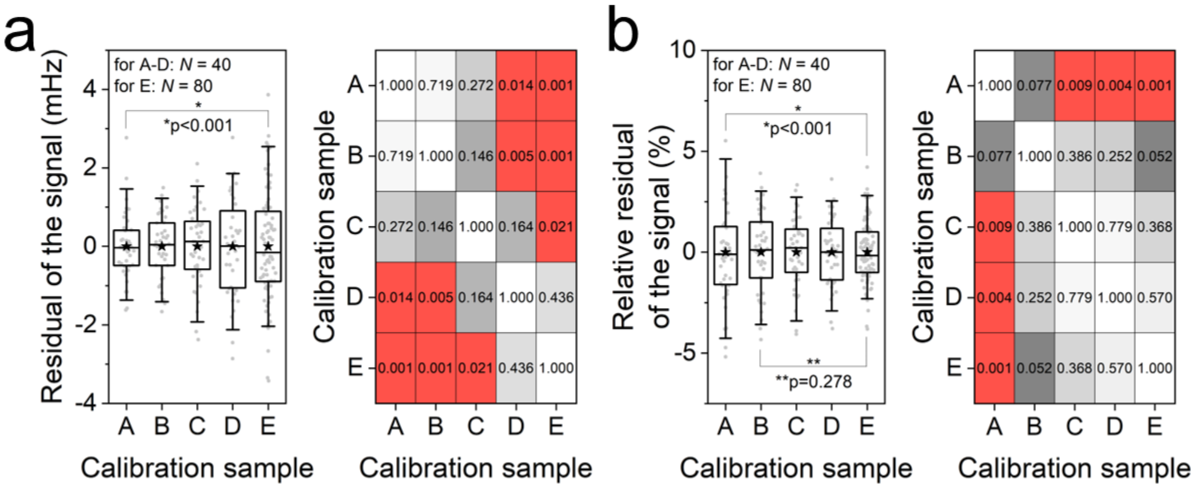

Figure S4. The (relative) residual of the signal per calibration sample. (a) Boxplot of the calculated residual of the measured signal in mHz per calibration sample. The boxplot indicates the mean (black star), 50th percentile (horizontal black line in box), 25th and 75th percentiles (top and bottom of box), and 5th and 95th percentiles (whiskers). The calculated residuals per measurement set are visualized (gray dots), where N = 40 for calibration samples A-D, and N = 80 for calibration sample E. Considering all calibration samples, the hypothesis that the mean of the calculated standard deviation for each calibration sample comes from the same distribution can be rejected (p < 0.001). The table shows that there are significant differences (red background) between the means of calculated standard deviation between multiple calibration samples. **(b)** Boxplot of the calculated relative residual of the measured signal in mHz per measurement set of five individual measurements on a calibration sample, grouped per calibration sample. The boxplot visualizes the same features as in panel a. Considering all calibration samples, the hypothesis that the mean of the calculated relative standard deviation for each calibration sample comes from the same distribution can be rejected (p < 0.001). Considering calibration samples B-E, the hypothesis

that the mean of the calculated relative standard deviation for these calibration samples comes from the same distribution cannot be rejected (p = 0.278). All reported p-values result from a Bartlett's test. An Anderson-Darling test indicates that a combined measurement set of all calibration samples (excluding calibration sample A) can be assumed to be normally distributed (p = 0.705, see Figure S6), as well as including all calibration samples (p = 0.107, see Figure S6). Therefore, for the Bartlett's test it was assumed that the calculated (relative) residual is normally distributed.

In Figure S5, using the measurement data of the example cartridge of Figure 3, the relative residuals calculated per calibration cycle (of in total eight consecutive calibration cycles) are visualized as a function of the calibration cycle, using the measurement data of all calibration samples. The p-values given in Figure S5 result from a Bartlett's test on the variance of the relative residual per calibration cycle, assuming normally distributed relative residuals.

Figure S5a shows a boxplot of the calculated relative residual per calibration cycle. In this Figure, the mean (black star), the 25th and 75th percentiles (black box), 50th percentile (horizontal black line in box), 5th and 95th percentiles (whiskers), and calculated relative residual per calibration cycle (gray dots) are visualized. Considering all calibration cycles, the hypothesis that the variances of the calculated relative residual per calibration cycle are the same is rejected (p = 0.015); this indicates that there is a concentration-dependency of the variance. However, by excluding calibration cycles 7 and 8, the hypothesis that the variances of the calculated relative residual per calibration cycle are the same, cannot be rejected (p = 0.266). Therefore, it was assumed that the concentration imprecision is largely time-independent at long as the cartridge is not used for 10 hours or more (*cf.* Figure S1 and Figure 3a). After this period, the concentration imprecision is increasing, probably caused by the long term changes of the sensor (see Supporting Information 1), which results in a smaller dynamic range of the signal.

Figure S5b gives the p-value from all possible combinations of comparing the calibration cycles. The p-values that result from a Bartlett's test show that there might only be a difference between the variance of the calculated relative residual in calibration cycle 3 and all other calibration cycles. To show the possibility of a type I error (false-positive), calibration cycle 3 has been used as an example in Figure 3.

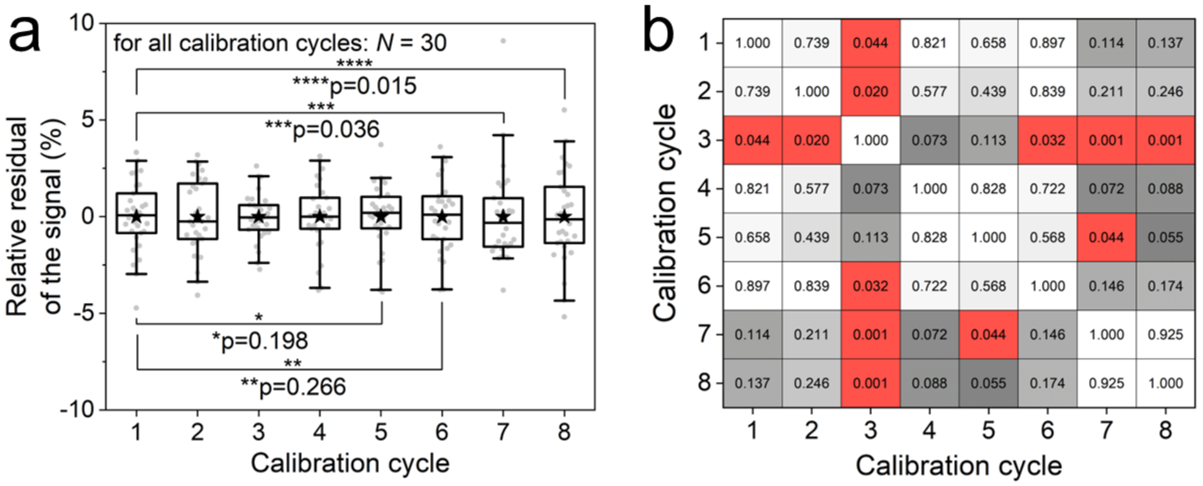

Figure S5. The relative residual of the measured signal per calibration cycle calculated per measurement set of five repeated measurements. (a) Boxplot of the calculated relative residual of the measured signal in mHz, grouped per calibration cycle. The boxplot indicates the mean (black star), 50th percentile (horizontal black line in box), 25th and 75th percentiles (top and bottom of box), and 5th and 95th percentiles (whiskers). The calculated

relative residuals are visualized (gray dots), where $N = 30$ for all calibration cycles. Considering all calibration samples, the hypothesis that the variance of the calculated relative residual for each calibration sample comes from the same distribution can be rejected ($p = 0.015$). Considering calibration cycles 1-6, the hypothesis that the variance of the calculated relative residual for these calibration samples comes from the same distribution cannot be rejected ($p = 0.266$). All reported *p*-values result from a Bartlett's test. **(b)** Table with *p*-values that results from a Bartlett's test which shows that there might only be a difference between the variance of the calculated relative residual in calibration cycle 3 and all other calibration cycles.

In conclusion, the signal and concentration imprecision are best quantified using relative residuals, since the distribution of the relative residuals appears to be normally distributed, and its mean and width do not depend on concentration or on time. This allows for combining measurement data to obtain a high statistical power and allows for subsequent statistical analysis.

Distributions of the relative residuals of the signal and concentration

Figure S6 shows histograms of the relative residual of the signal, using the same dataset as presented in Figures S4-S5. It was found that the relative residual of the signal was indeed normally distributed, both when including all data ($p = 0.107$, Anderson-Darling test) and when excluding calibration sample A ($p = 0.705$, Anderson-Darling test).

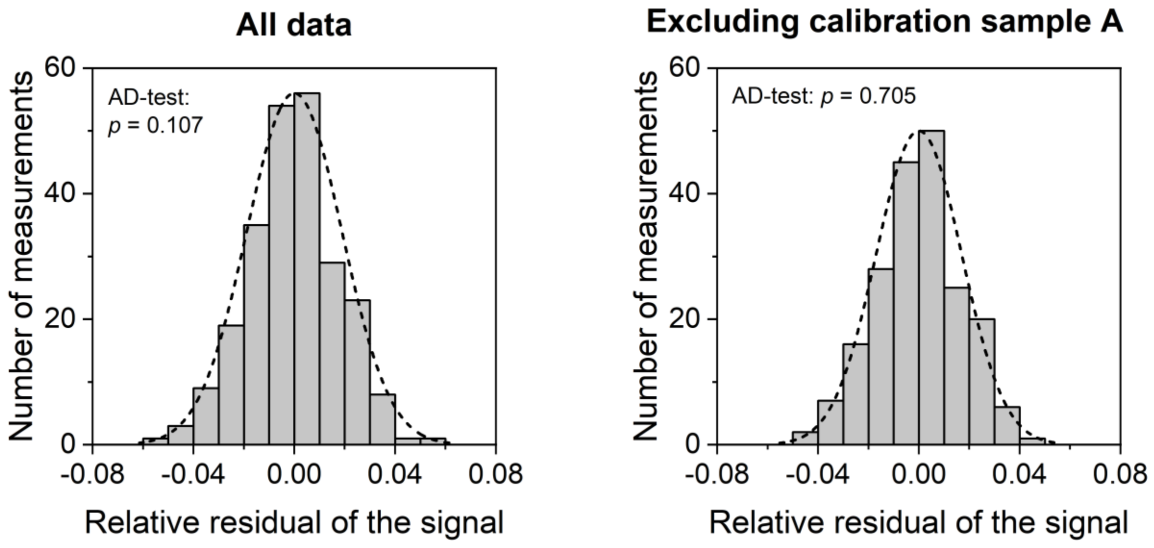

Figure S6. Histograms of the relative residual of the measured signal per calibration cycle calculated per measurement set of five repeated measurements. Right: histogram of the relative residual of the measured signal, for all data (left) and for the data excluding calibration sample A (right). For both histograms, the data appears to be normally distributed ($p = 0.107$, and $p = 0.705$, Anderson-Darling test).

In Figure S7, the boxplots and histograms of the relative residual of the concentration are given, using the same dataset as presented in Figures S4-S5. From the boxplot it can be concluded that there is a strong concentration dependency of the relative residual of the concentration, where a low and high GA concentration (*i.e.*, calibration samples A and E) have a larger variance compared to calibration samples with a GA concentration around the EC50 (*i.e.,* calibration sample C), see also for example Figure 3c. From the histograms it can be concluded that the relative residuals are normally distributed ($p = 0.138$, $p = 0.100$, $p = 0.102$,

p = 0.729, *p* = 0.561, Anderson-Darling test, for calibration samples A, B, C, D, and E respectively).

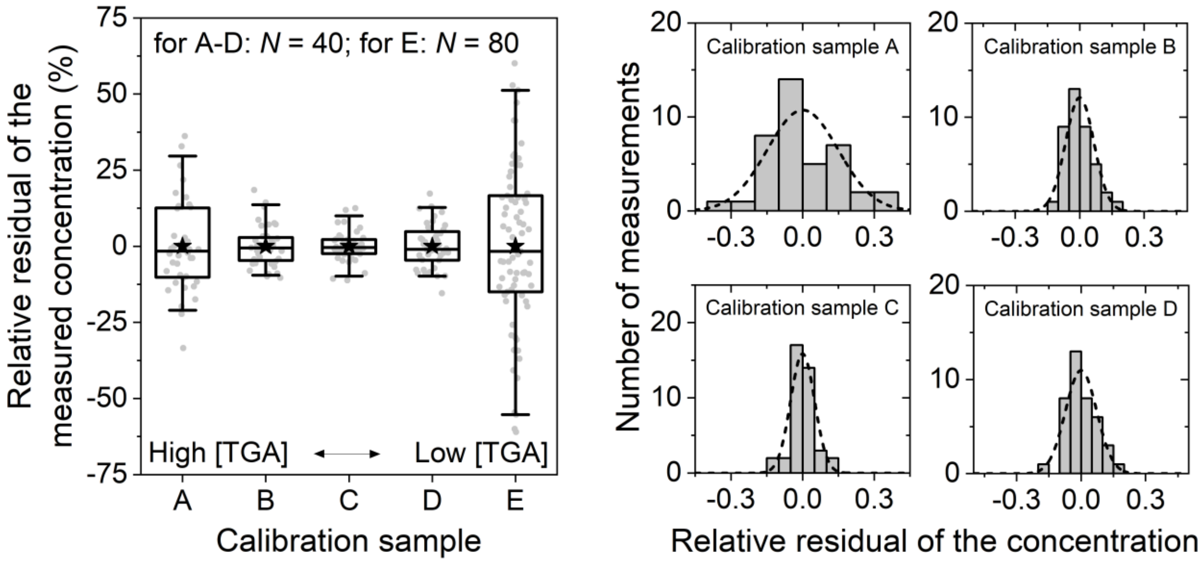

Figure S7. Boxplots and histograms of the relative residual of the measured concentration per calibration sample, calculated per measurement set of five repeated measurements. Left: boxplot of the relative residual of the measured concentration, grouped per calibration sample. There appears to be a strong concentration dependency of the shape of the distribution on the calibration sample. Right: histogram of the relative residual of the measured concentration, for calibration samples A-D. All shown histograms appear to be normally distributed ($p = 0.138$, $p = 0.100$, $p = 0.102$, $p = 0.729$, $p = 0.561$, Anderson-Darling test, for calibration samples A, B, C, D, and E respectively).

3. How the concentration imprecision depends on concentration

The concentration imprecision is defined as the standard deviation of a measurement set resulting from fitting the relative residual distribution of the measured data. The signal standard deviation results from the residual distribution of the signal, referred to as the absolute signal error s_s. The concentration standard deviation results from the residual distribution of the concentration, referred to as the absolute concentration error s_c.

Theoretically, the absolute concentration error can be calculated from the absolute signal error using Equation S3.

$$s_c = s_s \cdot \left|\frac{\delta[GA]}{\delta A}\right| \tag{S3}$$

where s_c is the absolute concentration error, s_s the absolute signal error, and $\frac{\delta[GA]}{\delta A}$ the inverse of the derivative of Equation 1. Supporting Information 2 showed that the width of the distribution of *absolute* residuals depends on concentration, but not the width of the *relative* residuals. Therefore, Equation S3 is rewritten to:

$$\widetilde{s_c} = \frac{A}{[GA]}\widetilde{s_s} \cdot \left|\frac{\delta[GA]}{\delta A}\right| \tag{S4}$$

where $\widetilde{s_c} = \frac{s_c}{[GA]}$ is the relative concentration error, *i.e.*, the concentration imprecision, and $\widetilde{s_s} = \frac{s_s}{A}$ is the relative signal error, *i.e.*, the signal imprecision. Combining Equations 1 and S4 results in Equation S5.

$$\widetilde{s_c} = \widetilde{s_s}\left|\left(p_1[GA]^n + \frac{p_2}{[GA]^n} + p_3\right)\right| \tag{S5}$$

where p_1, p_2, and p_3 are constants. For visualization purposes, in this paper the concentration imprecision curves are visualized using a double logarithmic scale, see Figure S8. Using this double logarithmic scale, the concentration imprecision as a function of concentration resembles a second-order polynomial. Deviations are seen for concentrations far below and far above the EC_{50}. The polynomial approximation appears to be valid in the concentration range of the calibration samples, *i.e.,* approximately $0.1 \cdot EC_{50} < [GA] < 10 \cdot EC_{50}$.

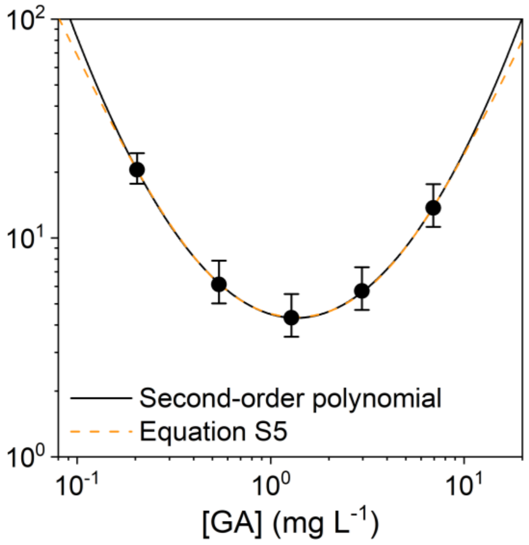

Figure S8. Concentration imprecision as a function of GA concentration, fitted with a second-order polynomial (black line) and with Equation S5 (orange dashed line). The second-order polynomial and Equation S5 show good agreement for GA concentrations close to the EC_{50}, *i.e.,* the minimum of the concentration imprecision curve. The example data is data from Figure 3c.

4. Quantifying BPM sensor imprecision using measurement samples

The measurement imprecisions were quantified mostly using calibration samples, *i.e.,* diluted, mixed potato fruit juice (PFJ) samples, see Figures 3 and 4. The use of well-defined calibration samples allows one to make clear distinctions between imprecision (random errors) and accuracy (systematic errors). Furthermore, this provides the opportunity to obtain a high statistical power by combining measurement sets. However, this raises the question if the obtained imprecision values are also applicable to other PFJ samples that may have slightly different matrix compositions. Here, we study if there are differences between imprecision values determined using calibration samples and determined using other PFJ samples. Details of the sample pretreatment and measurement procedures are given at the end of this Section.

The BPM sensor imprecision is calculated as follows, exemplified with the data in Table S1. The same procedure applies to calculating the BPM sensor imprecision from measurement samples and from calibration samples.

1. Calculate the mean concentration over all repeated measurements, for each replicate sample. For Table S1, this results in five mean concentrations per PFJ sample per cartridge, as there are five replicate samples measured per PFJ sample per cartridge.

2. Determine the relative residuals using the mean concentrations and the measured concentrations. For Table S1, this yields 15 relative residuals per PFJ sample per cartridge, since there are five replicate samples and each replicate sample has 3 repeated measurements.

3. Fit all relative residuals per PFJ sample per cartridge with a normal distribution. The standard deviation and the standard error of the fit are then reported as the concentration imprecision for that particular PFJ sample on that particular cartridge. For Table S1, this yields nine concentration imprecision values, since three PFJ samples were each measured on three different cartridges (*cf.* Figure S9).

Figure S9 visualizes the BPM sensor imprecision calculated using calibration samples (gray dots), with corresponding standard error (gray error bars), and measurement samples (black dots), with corresponding standard error (black error bars) as a function of GA concentration. The measurement data of all individual measurements are given in Table S1. The reported BPM sensor imprecision resulting from the second-order polynomial fits (gray lines and orange stars) is 6.3% (5.8%-6.9%, SE), 3.8% (2.4%-6.3%, SE), and 3.1% (2.7%-4.2%, SE) for three cartridges A-C, respectively. The SE of the BPM sensor imprecision (horizontal orange dashed lines) overlaps with the BPM sensor imprecision calculated using measurement samples. For cartridge B, there appears to be a difference between the BPM sensor imprecision calculated using calibration samples and measurement samples, which is probably due to a large spread in the BPM sensor imprecision per calibration sample; this results in the large SE of the reported BPM sensor imprecision. Therefore, it was concluded that there is no significant difference between BPM sensor imprecision calculated using calibrations samples, or calculated using measurement samples.

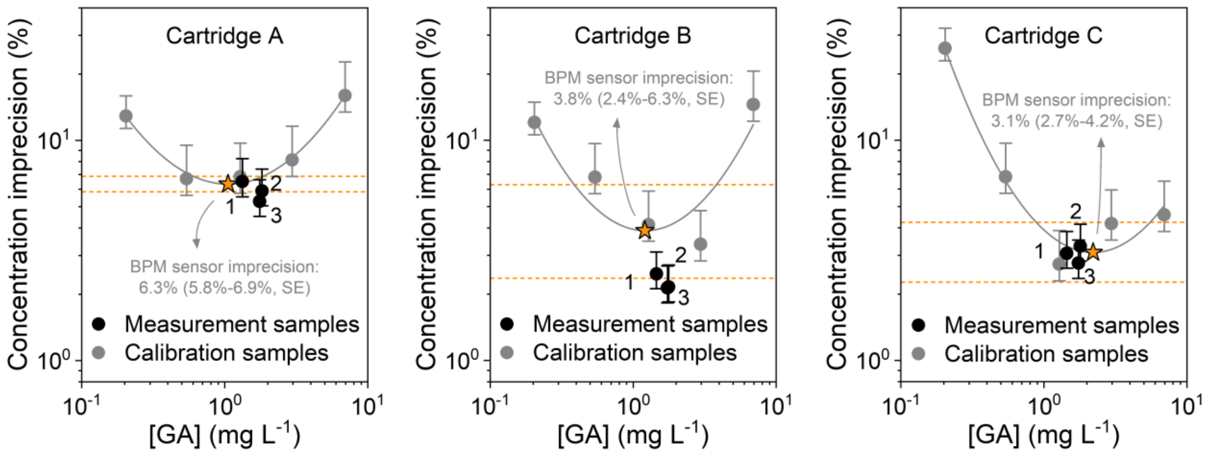

Figure S9. Quantification of BPM sensor imprecision using calibration samples and measurement samples. The BPM sensor imprecision calculated using calibration samples (gray dots) with corresponding standard error (gray error bars), and a second-order polynomial fit (gray line) as a function of GA concentration, and the BPM sensor imprecision calculated using measurement samples as a function the measured GA concentration (black dots) with corresponding standard error (black error bars), for cartridges A-C. The reported BPM sensor precisions of the three cartridges (orange stars) are 6.3% (5.8%-6.9%, SE), 3.8% (2.4%-6.3%, SE), and 3.1% (2.7%-4.2%, SE). For all cartridges, the standard error of the BPM sensor imprecision calculated using calibration samples (horizontal dashed orange line), largely overlaps with the BPM sensor imprecision calculated using measurement samples. All data were measured at location B.

All measurement data for the quantification of the BPM sensor imprecision using measurement samples can be found in Table S1.

Table S1. Measurement data of repeated measurements on three PFJ samples on three cartridges for BPM sensor imprecision quantification. Measured GA concentration in mg L^{-1} of all measurement samples. Per PFJ sample, 15 measurement samples were prepared (for each PFJ sample, five measurement samples per cartridge were measured), of which each measurement sample was measured thrice; measurement sample number (first column, five per PFJ sample) and repeated measurement number (second column, three per measurement sample. This results in 45 measurement samples (of three PFJ samples) and 135 measurement readings of which one measurement could not be measured (indicated by *N/A*) due to fluidic transportation problems (possibly air bubbles were present in the sample) or software errors, and one measurement was regarded as an outlier (indicated by *), resulting in N = 133. All data were measured at location B.

		**Cartridge A**			**Cartridge B**			**Cartridge C**		
Repl. Sam.	*Rep. meas.*	*Sample nr.*			*Sample nr.*			*Sample nr.*		
		1	*2*	*3*	*1*	*2*	*3*	*1*	*2*	*3*
1	1	1.32	1.87	1.73	1.44	1.64	1.62	1.48	1.82	1.80
1	2	1.46	1.96	1.89	1.41	1.68	1.65	1.46	1.78	1.71
1	3	1.28	1.94	1.89	1.42	1.75	1.59	1.50	1.74	1.72
2	1	1.36	1.61	1.88	1.35	1.71	1.65	1.31	1.87	1.76
2	2	1.39	1.69	1.77	1.35	1.76	1.70	1.45	1.81	1.77
2	3	1.46	1.83	1.72	1.42	1.75	1.69	1.40	1.78	1.73
3	1	1.33	1.78	1.38	1.43	1.89	1.85	1.46	1.79	1.74
3	2	1.38	1.77	1.60	1.55	1.80	1.85	1.47	1.75	1.88
3	3	0.77*	1.89	1.61	1.45	1.78	1.88	1.55	1.75	1.78
4	1	1.32	1.80	1.76	1.50	1.80	1.69	1.44	1.67	1.80
4	2	1.39	1.81	1.83	1.50	1.83	1.73	1.38	1.88	1.77
4	3	1.20	1.89	1.63	1.49	1.74	1.75	1.48	1.91	1.71

5	1	1.05	1.66	1.75	1.54	1.80	1.79	1.39	1.87	1.56
5	2	1.33	2.11	1.99	1.51	1.81	1.93	1.40	1.76	1.69
5	3	1.32	1.74	1.89	1.44	1.85	1.76	1.50	1.79	*N/A*

Figure S10 shows the measured concentration of PFJ sample numbers 1-3 (*i.e.,* the averaged concentration of five replicate measurement samples) which have been measured on three cartridges on three different measurement days. It was found that the concentration per sample measured over all cartridges is quite similar. Using all measurement data, it is found that the GA concentration of sample 1 is 141±9 mg L^{-1}, sample 2 is 216±11 mg L^{-1}, and sample 3 is 245±16 mg L^{-1}, where the reported error is the sample standard deviation of all measurements in Table S1.

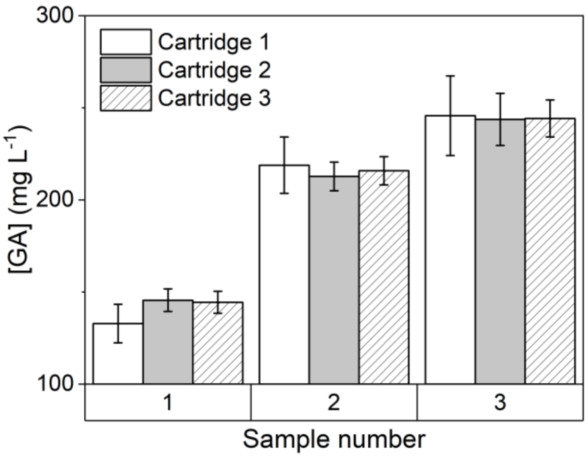

Figure S10. Measured GA concentration for PFJ sample numbers 1, 2 and 3, using three cartridges, measured on three measurement days. Measured GA concentration of PFJ sample numbers 1, 2 and 3, measured on cartridge A (white bar), cartridge B (light gray bar), and cartridge C (striped bar) with corresponding sample standard deviation (black error bars). Using the measurement data of all three cartridges, it was found that the GA concentration of sample 1 is 141±9 mg L^{-1}, sample 2 is 216±11 mg L^{-1}, and sample 3 is 245±16 mg L^{-1}, where the reported error is the sample standard deviation.

Materials and methods for the quantification of BPM sensor imprecision using measurement samples

Sample preparation: A total of three potato fruit juice (PFJ) samples were prepared at Avebe by manual grating of potatoes of several varieties. The PFJ samples were stored at -20°C until further use. Five replicates per PFJ sample were prepared, resulting in a total of 15 measurement samples, using the following steps: per PFJ sample, the sample was centrifuged at 6,000 × g for 5 min using a tabletop spinner (Eppendorf MiniSpin) after which the supernatant liquid was diluted in bulk (100x, 120x, and 140x for sample 1, 2, and 3 respectively) in PBS with an additional 500 mM NaCl. The centrifuged and diluted measurement samples were stored at -20°C until further use.

Measurement procedure: Per cartridge, five replicate measurement samples per PFJ sample were selected and measured in a single run using a randomized order. Three repeated measurements were performed on a single measurement sample. The relative residuals per measurement were calculated using the mean of the three individual measurements, which yields 15 relative residuals per PFJ sample per cartridge. The measurement results are given in Table S1.

5. Sample-to-sample imprecision

The sample-to-sample imprecision was quantified separately for sample dilution only (Figure S11) and for sample centrifugation and dilution (Figure S12) within the applied sample pretreatment protocol (see Materials and Methods). This has been done to study if dilution or centrifugation is the main source of variation in the sample-to-sample imprecision. Details of the sample pretreatment and measurement procedures are given at the end of this Section.

Sample-to-sample imprecision - dilution only

14 samples were measured on three cartridges: six samples on cartridge D, six samples on cartridge E, and two samples on cartridge F. Since we are interested in the sample-to-sample imprecision, it is important to take the cartridge-to-cartridge variation into account (see Figure 3d) since multiple cartridges were used in this analysis. Therefore, per cartridge, the BPM sensor imprecision is compared to the observed concentration imprecision per sample.

In Figure S11, the measurement results of three cartridges are given using the BPM sensor imprecision as a function of GA concentration (gray dots), the corresponding standard error (gray error bars), and a second-order polynomial fit (gray line). The BPM sensor precision (orange star) results from the minimum imprecision found by using the fit. The found BPM sensor imprecision per cartridge is given in the Figure along with the corresponding SE for prediction. The concentration imprecision per sample for the measured GA concentration (black dots) is given along with the corresponding sample number, for dilution only as sample pretreatment steps. Almost all observed concentration imprecisions are on or above the BPM sensor imprecision curve; this is expected since for sample-to-sample imprecision the included sources of variations are at least the same as the included sources of variation for the BPM sensor imprecision. Per cartridge, the absolute difference between the BPM sensor imprecision and the mean observed concentration imprecision for all samples measured on that cartridge, is given as calculated using Equation 3, and results in values between approximately 3%pt. and 5%pt. The average BPM sensor precision of all three cartridges was found to be 4.6% (3.6-5.9%, SE), which is the data shown in Figure 4c (top left, black line).

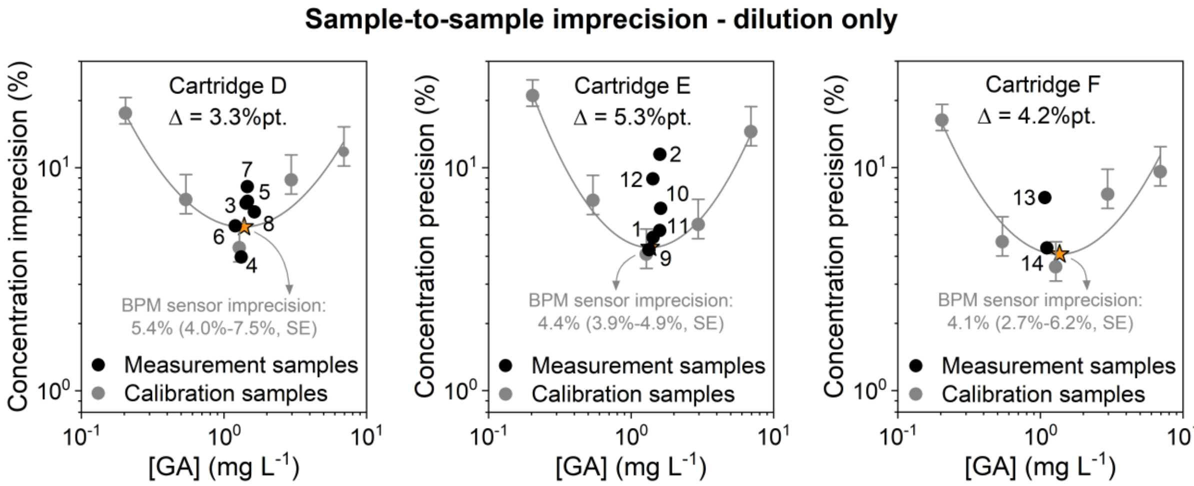

Figure S11. Quantification of sample-to-sample imprecision including dilution only. The BPM sensor imprecision calculated using calibration samples (gray dots) with corresponding standard error (gray error bars), and a second-order polynomial fit (gray line) as a function of GA concentration, and the sample-to-sample imprecision (including dilution only) calculated using measurement samples as a function of the mean measured GA concentration (black dots), for cartridges D-F. The reported BPM sensor imprecision of the three cartridges

(orange stars) is 5.4% (4.0%-7.5%, SE), 4.4% (3.9%-4.9%, SE), and 4.1% (2.7%-6.2%, SE). The difference between the mean sample-to-sample imprecision and the BPM sensor precision is 3.3%pt., 5.3%pt., and 4.2%pt, calculated using Equation 3. All data were measured at location B.

All measurement data for the quantification of the sample-to-sample imprecision including dilution only, are listed in Table S2.

Table S2. Measurement data of repeated measurements on 14 PFJ samples on three cartridges for sample-to-sample imprecision quantification, including dilution only. Measured GA concentration in mg L^{-1} of all measurement samples. Per PFJ sample, five measurement samples were prepared, of which each measurement sample was measured thrice; measurement sample number (first column, five per PFJ sample) and repeated measurement number (second column, three per measurement sample). This results in 70 measurement samples (of 14 PFJ samples) and 210 measurement readings of which six measurements could not be measured (indicated by *N/A*) due to fluidic transportation problems (possibly air bubbles present in the sample) or software errors, and one measurement was regarded as an outlier (indicated by *), resulting in *N* = 203. Data were measured at location B.

Repl. Sam.	*Rep. meas.*	*Sample nr.*													
		1	*2*	*3*	*4*	*5*	*6*	*7*	*8*	*9*	*10*	*11*	*12*	*13*	*14*
1	1	1.39	1.74	1.65	1.25	1.42	1.16	1.30	1.42	1.45	1.45	1.48	1.29	1.17	1.11
1	2	1.46	1.77	1.48	1.42	1.42	1.24	1.39	1.50	1.37	1.49	1.69	1.52	1.09	1.11
1	3	1.40	1.72	1.48	1.38	1.50	1.21	1.37	1.58	1.22	1.52	1.6	1.46	1.12	1.16
2	1	1.38	1.63	1.34	1.31	1.44	1.14	1.40	1.76	1.29	1.54	1.59	1.55	1.14	0.63*
2	2	1.55	1.94	1.43	1.34	1.29	1.06	1.32	1.72	1.30	1.54	1.64	1.70	1.27	1.04
2	3	1.46	1.90	1.45	1.35	1.39	*N/A*	1.36	1.73	1.28	1.48	*N/A*	*N/A*	0.95	1.09
3	1	1.43	1.39	1.33	1.37	1.47	1.32	1.39	1.72	1.37	1.65	1.65	1.48	1.00	1.05
3	2	1.43	1.53	1.37	1.30	1.45	1.26	1.46	1.66	1.32	1.64	1.65	1.37	1.04	1.14
3	3	1.55	1.46	1.44	1.30	1.54	1.26	1.47	1.57	1.34	1.63	1.61	1.55	0.98	1.19
4	1	1.36	1.44	1.36	1.27	1.48	1.15	1.59	1.62	1.30	1.64	1.47	1.23	1.05	1.13
4	2	1.45	1.63	1.28	1.26	1.68	1.18	1.37	1.64	1.30	1.81	1.63	1.35	1.10	1.14
4	3	1.47	1.49	1.60	1.31	1.68	1.19	1.54	1.69	1.36	1.72	1.72	1.43	1.06	1.18
5	1	1.40	1.45	1.46	1.39	1.39	1.17	1.68	*N/A*	1.38	1.73	1.46	1.34	1.07	1.05
5	2	1.27	1.41	1.41	1.25	1.38	1.25	1.60	*N/A*	1.4	1.66	1.51	1.31	1.04	1.10
5	3	1.42	1.42	1.37	1.29	1.42	1.24	1.62	*N/A*	1.37	1.72	1.58	1.37	1.04	1.08

Sample-to-sample imprecision - centrifugation and dilution

14 samples have been measured on three cartridges: five PFJ samples on cartridge G, six PFJ samples on cartridge H, and three PFJ samples on cartridge I. Again, since we are interested in the sample-to-sample imprecision only, it is important to take into account the cartridge-to-cartridge variation (see Figure 3d) since multiple cartridges were used in this analysis. Therefore, per cartridge, the BPM sensor imprecision is compared to the observed concentration imprecision per sample.

In Figure S12, the measurement results of three cartridges are given using the BPM sensor imprecision as a function of GA concentration (gray dots) and the corresponding standard error (gray error bars), and a second-order polynomial fit (gray line). The BPM sensor precision (orange star) results from the minimum imprecision found by using the fit. The found BPM sensor imprecision per cartridge is given in the Figure along with the corresponding SE for

prediction. The sample-to-sample concentration imprecision per sample for the measured GA concentration (black dots) is given along with the corresponding sample number, for centrifugation and dilution as sample pretreatment steps. All observed concentration imprecisions are on or above the BPM sensor imprecision curve, which is as expected since for sample-to-sample imprecision the included sources of variations are at least the same as included for the BPM sensor imprecision. Per cartridge, the difference between the BPM sensor imprecision and the mean observed concentration imprecision for all samples measured on that cartridge, is given as calculated using Equation 3, and results in values between approximately 4%pt. and 5%pt. The average BPM sensor precision of all three cartridges was found to be 3.7% (3.6%-3.9%, SE), which is the data shown in Figure 4c (bottom left, black line).

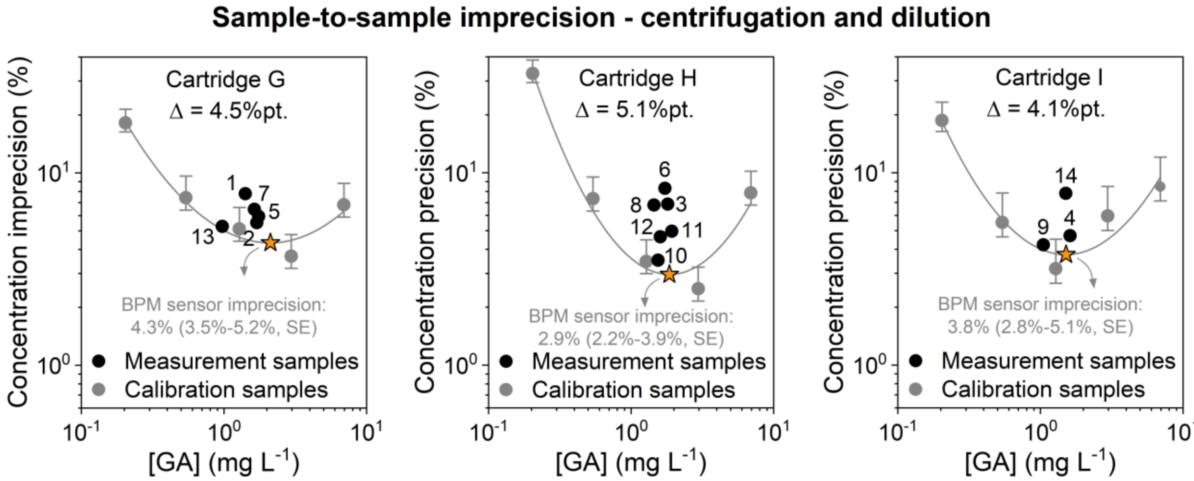

Figure S12. Quantification of sample-to-sample imprecision including centrifugation and dilution. The BPM sensor calculated using calibration samples (gray dots) with corresponding standard error (gray error bars), a second-order polynomial fit (gray line) as a function of GA concentration, and the sample-to-sample imprecision (including centrifugation and dilution) calculated using measurement samples as a function of the mean measured GA concentration (black dots) with corresponding standard error (black error bars), for cartridges G-I. The reported BPM sensor imprecision of the three cartridges (orange stars) is 4.3% (3.5%-5.2%), 2.9% (2.2%-3.9%), and 3.8% (2.8%-5.1%). The difference between the mean sample-to-sample imprecision and the BPM sensor precision is 4.5%pt., 5.1%pt., and 4.1%pt. All data were measured at location B.

All measurement data for the quantification of the sample-to-sample imprecision including centrifugation and dilution are listed in Table S3.

Table S3. Measurement data of repeated measurements on 14 PFJ samples on three cartridges for sample-to-sample imprecision quantification, including centrifugation and dilution. Measured GA concentration in mg L^{-1} of all measurement samples. Per PFJ sample, five measurement samples were prepared, of which each measurement sample was measured thrice; measurement sample number (first column, five per PFJ sample) and repeated measurement number (second column, three per measurement sample). This results in 70 measurement samples (of 14 PFJ samples) and 210 measurement readings of which eight measurements could not be measured (indicated by *N/A*) due to fluidic transportation problems (possibly air bubbles present in the sample) or software errors, and three measurements were regarded as an outlier (indicated by *), resulting in N = 199. Data were measured at location B.

Meas. Sam.	*Rep. meas.*	*Sample nr.*													
		1	*2*	*3*	*4*	*5*	*6*	*7*	*8*	*9*	*10*	*11*	*12*	*13*	*14*
1	1	1.32	1.66	1.77	1.53	1.64	1.71	1.65	1.42	1.11	1.54	2.04	1.61	0.96	1.67
1	2	1.37	1.68	1.78	1.51	1.72	1.80	1.67	1.39	1.04	1.51	2.02	1.55	1.02	1.76
1	3	1.39	1.68	1.67	*N/A*	1.78	1.72	1.55	1.43	1.02	1.58	2.05	1.61	0.99	1.42
2	1	1.26	1.57	1.64	1.61	1.68	1.91	1.48	1.51	0.97	1.53	1.80	1.50	1.01	1.47
2	2	1.26	1.71	1.64	1.62	1.88	1.61	1.52	1.51	1.06	1.48	1.89	1.53	0.99	1.41
2	3	1.31	1.82	1.80	1.63	1.64	1.75	1.56	1.66	1.02	1.53	1.80	1.59	0.96	1.37
3	1	1.37	1.63	2.05	1.66	1.75	1.70	1.69	1.47	0.98	1.45	1.90	1.69	1.01	1.52
3	2	1.38	1.75	1.94	1.71	1.73	1.58	1.77	1.33	1.03	1.52	1.84	1.74	1.08	1.41
3	3	1.52	1.66	1.84	1.62	2.01	1.58	1.44	1.35	1.03	1.61	1.93	1.63	0.92	1.37
4	1	1.59	1.93	1.92	1.55	1.79	1.92	1.79	*N/A*	1.14	1.61	2.01	1.70	1.03	1.55
4	2	1.45	1.82	1.92	1.50	1.81	1.92	1.70	*N/A*	1.10	1.64	2.07	1.66	0.92	1.60
4	3	1.60	1.65	1.85	1.62	1.72	1.91	1.67	*N/A*	1.05	1.55	1.90	1.61	0.98	1.57
5	1	1.44	1.69	1.85	1.59	1.84	1.44	1.75	*N/A*	1.07	1.62	4.28*	1.48	0.92	1.38
5	2	1.53	1.63	1.67	1.70	1.61	1.66	1.62	*N/A*	1.05	1.56	5.22*	1.65	0.91	1.56
5	3	1.38	1.63	*N/A*	1.75	1.70	1.74	1.65	*N/A*	1.05	1.50	3.13*	1.55	0.92	1.57

Correction for cartridge-to-cartridge variation

For all cartridges used in the study for sample-to-sample imprecision for dilution only, it was found that the BPM sensor imprecision is 4.6% (3.6-5.9%, SE). For all cartridges used in the study for sample-to-sample imprecision for centrifugation and dilution, it was found that the BPM sensor imprecision is 3.7% (3.6%-3.9%, SE). On average, the absolute difference in BPM sensor precision between the two sets of cartridges is $\sqrt{4.6^2 - 3.7^2} = 2.7\%$, which is most likely caused by cartridge-to-cartridge variation (*cf.* Figure 3d).

In Figure S13 the relative residuals are shown for the studies on sample-to-sample imprecision for dilution only (uncorrected and corrected for cartridge-to-cartridge variation) and on sample-to-sample imprecision for centrifugation and dilution. The sample-to-sample imprecision when using only the measurement set including dilution only, was found to be 6.6% (6.3%-7.0%, SE). The sample-to-sample imprecision when using only the measurement set including centrifugation and dilution, was found to be 5.9% (5.6%-6.2%, SE). The difference in the variance of these two distributions does not appear to be significantly different (p = 0.100, Bartlett's test, assuming a normal distribution). However, as mentioned earlier, the cartridge-to-cartridge variation results in an offset between the two measurement sets which has to be corrected for. A crude correction method for this would be to subtract the contribution of cartridge-to-cartridge variation. Using this approach, the sample-to-sample imprecision for the measurement set including dilution only would result in $\sqrt{6.6^2 - 2.7^2} = 6.0\%$, rather than 6.6%. Since the sample-to-sample imprecision is based on the width of the distribution of the

observed relative residuals, a correction factor of 0.91 (i.e., 6.0%/6.6%) can be applied to the relative residuals of the measurement set including dilution only. Combining measurement data of Figures S12-S13 results in the boxplot visualized in Figure 4c. The sample-to-sample imprecision when using only the measurement set for dilution only, after the correction was applied, was found to be 5.9% (5.7%-6.2%, SE). This is comparable to the sample-to-sample imprecision for centrifugation and dilution, since the difference of the variance between the measurement set for dilution only, corrected for cartridge-to-cartridge variation, and centrifugation and dilution, does not appear to be significant (p = 0.751, Bartlett's test, assuming a normal distribution).

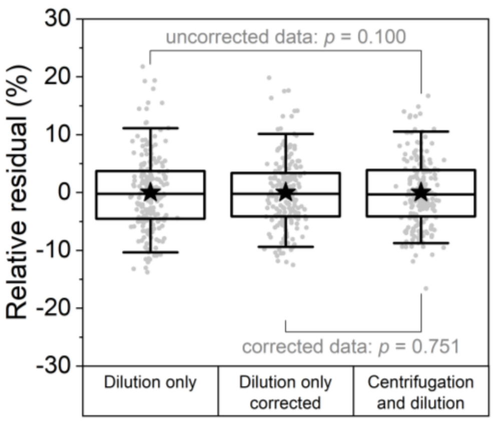

Figure S13. Comparison of the distributions of the observed relative residuals in quantifying the sample-to-sample imprecision. The found sample-to-sample imprecision was found to be 6.6% (6.3%-7.0%, SE), 6.0% (5.7%-6.3%, SE), and 5.9% (5.7%-6.2%, SE) for dilution only, dilution only corrected for cartridge-to-cartridge variation, and for centrifugation and dilution (for all dilution only N = 203, and for centrifugation and dilution N = 199). The difference in the variance for dilution only and centrifugation and dilution does not appear to be significant (p = 0.100, Bartlett's test, assuming a normal distribution), as well as the difference in the variance for dilution only corrected and centrifugation and dilution (p = 0.751, Bartlett's test, assuming a normal distribution). The boxplots indicate the mean (black star), 50th percentile (horizontal black line in box), 25th and 75th percentiles (top and bottom of box), and 5th and 95th percentiles (whiskers).

PFJ samples 1-14 were measured twice: once for the study on the influence of dilution only, and once for the study on the influence of dilution and centrifugation. Therefore, the measured concentration can be compared. Figure S14 shows the correlation plot of the measurement data in Tables S2-S3. Based on these measurement results, there appears to be a good correspondence between the two studies in the measured GA concentration.

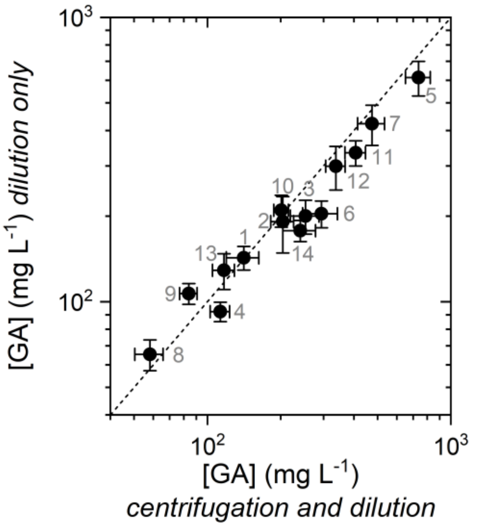

Figure S14. Correlation plot of the measurement data of 14 PFJ samples measured on three cartridges, for the dilution-only imprecision study and for the centrifugation-and-dilution imprecision study. Mean GA concentration (black dots) with corresponding mean reported BPM sensor imprecision (black error bars), for dilution only (see Table S2) and for centrifugation and dilution (see Table S3). The data is expected to be around $y = x$ (black dashed line). Data were measured at location B.

Material and methods for the quantification of sample-to-sample imprecision

Sample preparation (dilution-only imprecision study): 14 potato fruit juice (PFJ) samples were prepared at Avebe by manual grating of potatoes (of different varieties). The PFJ samples were stored at -20°C until further use. Five replicates per PFJ sample were prepared, resulting

in a total of 70 measurement samples, using the following steps: per PFJ sample, the sample was centrifuged (6000 × g for 5 minutes). After centrifugation, the supernatant liquid was divided over five Eppendorf tubes. Each measurement sample was diluted in PBS with an additional 500 mM NaCl, using dilution factors between 100x and 600x. The samples were stored at -20°C until further use.

Sample preparation (centrifugation and dilution imprecision study): 14 potato fruit juice (PFJ) samples were prepared as described above. However, *prior* to the centrifugation, per PFJ sample five replicates were divided over five Eppendorf tubes. The tubes were centrifuged (6000 × g for 5 minutes) and thereafter diluted in PBS with 500 mM NaCl. The samples were stored at -20°C until further use.

Measurement procedure: Calibration was performed with two sequential calibration cycles. Thereafter, a maximum of 15 measurement samples were measured in a randomized order. Thereafter the sensor was recalibrated with a single calibration cycle, and subsequently a maximum of 15 other samples were measured in a randomized order. The measurement procedure was repeated with a new cartridge until all samples were measured (three cartridges in total). In all cases, replicates of a single sample were measured on the same cartridge in the same cycle.

6. Dilution series of PFJ samples

Figure S15 shows dilution series measurements of three PFJ samples, using four dilutions per sample in PBS with an additional 500 mM NaCl. For all three PFJ samples, the data can be described well by $y = ax^n$, with $n = -1$, indicating that the PFJ matrix does not influence the measurement result. The error bars and data labels show the concentration imprecisions, which increase toward the maximum and minimum GA concentrations of the calibration samples (see dashed gray lines, see also Figure 3c). The prefactor a reveals the GA concentration in the undiluted PFJ sample, which is reported in Figure S15.

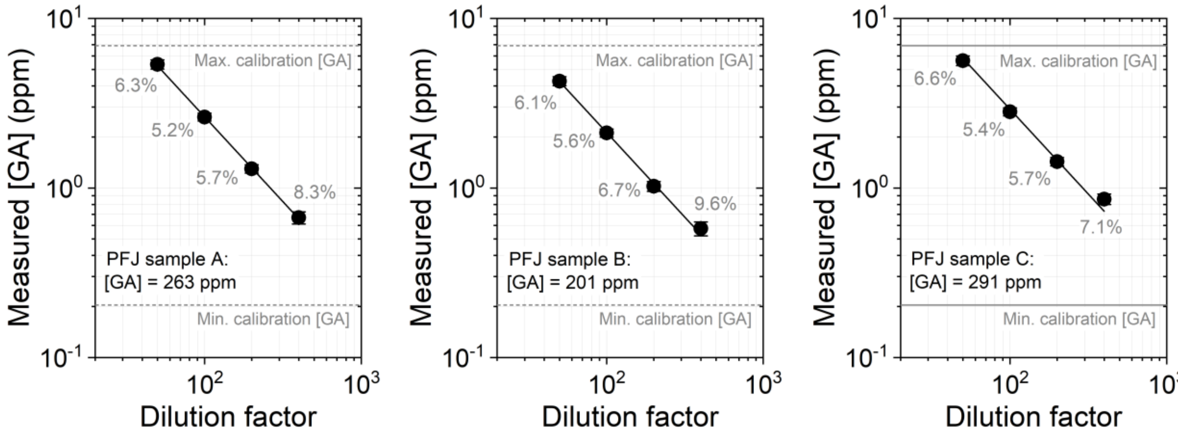

Figure S15. Dilution series of three PFJ samples. The measured GA concentration (black dots) is visualized as a function of the dilution factor. Data indicate the mean (*N* = 3). The error bars and data labels show the BPM sensor imprecision resulting from the calibration samples (black error bars, see also data labels). The maximum and minimum calibration limits are denoted by horizontal dashed lines. The data is fitted with $y = ax^n$ where $n = -1$ for all PFJ samples. GA concentrations of 263 ppm, 201 ppm, and 291 ppm were found for the example PFJ samples.

7. Comparison between measurements performed at locations A and B

Results from measurements on both locations A and B are reported throughout this paper. Here, we study if there are significant differences between the measurements performed at location A or B. Therefore, the baseline and background signals, decay rates, and signal and concentration BPM sensor imprecisions are compared in Figure S16.

In Figure S16a, the baseline signal A_{bl} and background signal A_{bg} resulting from the Hill equation fit (see Equation 1), are compared. The difference between the mean observed A_{bl} and A_{bg} measured on location A (*N* = 21) and location B (*N* = 26) are not significant (*p* = 0.183 and *p* = 0.115 respectively, one-way ANOVA, assuming a normal distribution). However, on average both A_{bl} and A_{bg} are slightly smaller for the measurements at location B. Therefore, the average measured signals are lower at location B than at location A. For the decay rate k_{decay}, resulting from Equation S1, two cartridges for location A and three cartridges for location B showed a (small) negative decay rate which means that the measured signal increases over time. These cartridges were excluded for their deviating behavior, resulting in *N* = 19 cartridges for location A, and *N* = 23 cartridges for location B. The difference between the mean observed k_{decay} measured at locations A and B is not significant (*p* = 0.125, one-way ANOVA, assuming a log-normal distribution).

Figure S16b shows the BPM sensor imprecision for both signal and concentration. The mean signal imprecision appears to be significantly different at locations A and B (*p* = 0.014, one-way ANOVA, assuming a log-normal distribution). The hypothesis for this difference is the lower average measured signal for the cartridges used at location A (see panel a), since for lower signals, sensor noise has a larger relative contribution to the observed imprecision. However, this significant difference is not observed in the concentration imprecision (*p* = 0.984, one-way ANOVA, assuming a log-normal distribution). A possible explanation is that other sources of variation are dominating the concentration imprecision rather than the slightly smaller average measured signal. Therefore, in this paper, it was assumed that that the BPM sensor concentration imprecision differences at location A and B were negligible.

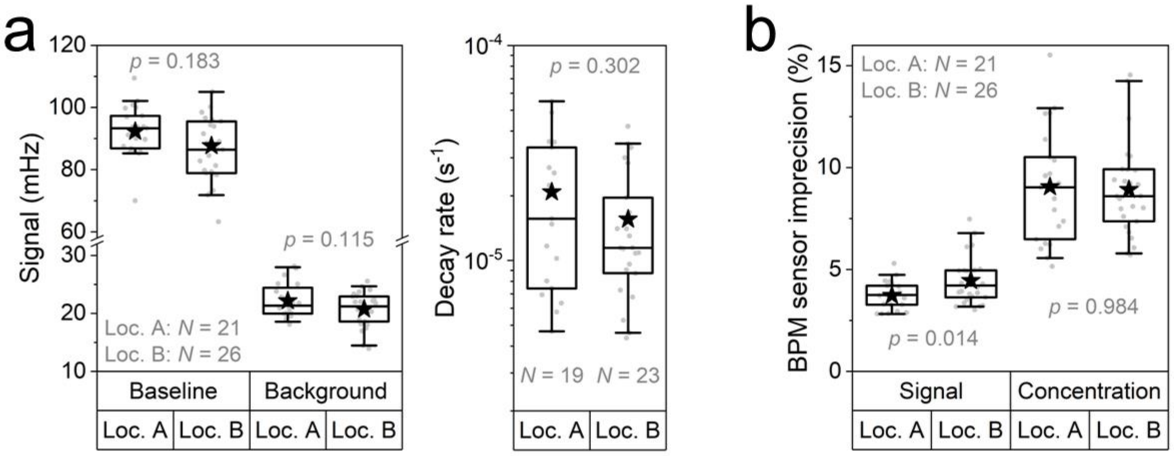

Figure S16. Comparison of measurements at locations A and B. (a) Comparison of (left) Hill Equation fit parameters baseline signal A_{bl} and background signal A_{bg} (see Equation 1), and (right) decay rate k_{decay} (see Equation S1) for all cartridges measured at location A (*N* = 21) and location B (*N* = 26). Both A_{bl} and A_{bg} do not appear to be significantly different for measurements at both locations (*p* = 0.183 and *p* = 0.115 respectively, one-way ANOVA, assuming a normal distribution). Also, k_{decay} does not appear to be significantly different for measurements at both locations (*p* = 0.302, one-way ANOVA, assuming a log-normal distribution). For the analysis of k_{decay}, two cartridges were excluded from the set of measurements at location A and three cartridges were

excluded from the set of measurements at location B since these cartridges showed a deviating decay characteristics. The boxplots indicate the mean (black star), 50th percentile (horizontal black line in box), 25th and 75th percentiles (top and bottom of box), and 5th and 95th percentiles (whiskers). **(b)** Comparison of the BPM sensor imprecision of the measured signal and concentration for all cartridges measured at location A (N = 21) and location B (N = 26). There appears to be a significant difference in the BPM sensor signal imprecision (p = 0.014, one-way ANOVA, assuming a log-normal distribution), while the BPM sensor concentration imprecision appears to be similar for measurements at both locations (p = 0.984, one-way ANOVA, assuming a log-normal distribution). The boxplot indicates the same features as described in panel a.

All PFJ samples were measured at both locations. Therefore, the measured concentrations can be compared. Figure S17 shows the correlation plot of the measurement data in Table S3 (location B) and the data measured at location A (data are not listed in a Table). Based on these measurement results, there appears to be a good correspondence between the GA concentrations measured at the two different locations, by different persons.

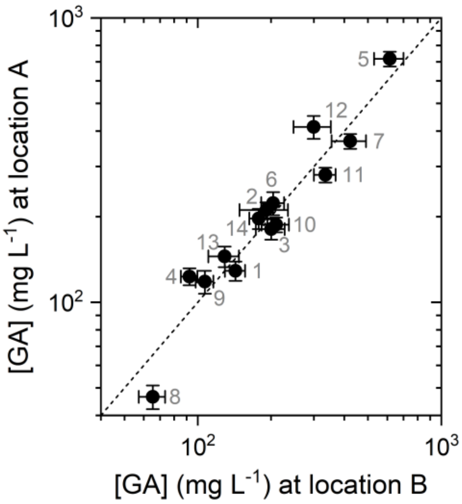

Figure S17. Correlation plot of the measurement data of 14 PFJ samples measured on three cartridges, measured at two different locations by different persons. Mean GA concentration (black dots) with corresponding mean reported BPM sensor imprecision (black error bars), for location A and for location B (see Table S2). The data is expected to be around y = x (black dashed line).

8. References

[1] Cajigas, S., de Jong, A.M., Yan, J., and Prins, M.W.J. Molecular origins of long-term changes in a competitive continuous biosensor with single-molecule resolution, *ACS Sens.*, 2024, **9**, 3520-3530.
